# Osteoarthritis-Related Degeneration Alters the Biomechanical Properties of Human Menisci Before the Articular Cartilage

**DOI:** 10.3389/fbioe.2021.659989

**Published:** 2021-05-06

**Authors:** Andreas M. Seitz, Felix Osthaus, Jonas Schwer, Daniela Warnecke, Martin Faschingbauer, Mirco Sgroi, Anita Ignatius, Lutz Dürselen

**Affiliations:** ^1^Institute of Orthopedic Research and Biomechanics, Center of Trauma Research Ulm, Ulm University Medical Center, Ulm, Germany; ^2^Department of Orthopedic Surgery, Universitäts- und Rehabilitationskliniken Ulm (RKU), Ulm University Medical Center, Ulm, Germany

**Keywords:** knee, meniscus, degeneration, instantaneous modulus, thickness, water content, equilibrium modulus, mapping

## Abstract

An exact understanding of the interplay between the articulating tissues of the knee joint in relation to the osteoarthritis (OA)-related degeneration process is of considerable interest. Therefore, the aim of the present study was to characterize the biomechanical properties of mildly and severely degenerated human knee joints, including their menisci and tibial and femoral articular cartilage (AC) surfaces. A spatial biomechanical mapping of the articulating knee joint surfaces of 12 mildly and 12 severely degenerated human cadaveric knee joints was assessed using a multiaxial mechanical testing machine. To do so, indentation stress relaxation tests were combined with thickness and water content measurements at the lateral and medial menisci and the AC of the tibial plateau and femoral condyles to calculate the instantaneous modulus (IM), relaxation modulus, relaxation percentage, maximum applied force during the indentation, and the water content. With progressing joint degeneration, we found an increase in the lateral and the medial meniscal instantaneous moduli (*p* < 0.02), relaxation moduli (*p* < 0.01), and maximum applied forces (*p* < 0.01), while for the underlying tibial AC, the IM (*p* = 0.01) and maximum applied force (*p* < 0.01) decreased only at the medial compartment. Degeneration had no influence on the relaxation percentage of the soft tissues. While the water content of the menisci did not change with progressing degeneration, the severely degenerated tibial AC contained more water (*p* < 0.04) compared to the mildly degenerated tibial cartilage. The results of this study indicate that degeneration-related (bio-)mechanical changes seem likely to be first detectable in the menisci before the articular knee joint cartilage is affected. Should these findings be further reinforced by structural and imaging analyses, the treatment and diagnostic paradigms of OA might be modified, focusing on the early detection of meniscal degeneration and its respective treatment, with the final aim to delay osteoarthritis onset.

## Introduction

Knee joint osteoarthritis (OA) is a prevalent and disabling disease that globally incurs increasing socioeconomic costs (Neogi, 2013). It has incidence and progression rates of approximately 2.5 and 3.6%, respectively (Cooper et al., 2000). While total knee replacement is a highly cost-effective and quality of life-improving approach to treat patients suffering from end-stage OA (CrossIII, Saleh et al., 2006; RuizJr., Koenig et al., 2013), the early detection and consequent treatment of early or pre-OA remain major challenges (Chu et al., 2012; Ryd et al., 2015). While dependencies between knee OA and meniscal degeneration have been previously described (Bhattacharyya et al., 2003; Hunter et al., 2006; Lohmander et al., 2007; Englund, 2009; Englund et al., 2009, 2012), it remains unknown as to which is the cause and which is the consequence of these entities. On the one hand, studies have shown an increased OA incidence in knees affected by meniscal injuries (Lohmander et al., 2007; Englund, 2009). On the other hand, degenerated knee joints also indicate degenerative changes of the menisci, including tears, macerations, and tissue loss (Bhattacharyya et al., 2003; Hunter et al., 2006; Englund et al., 2009), thereby leading to controversy in the treatment of knee joint OA, as summed up by Englund et al. (2009): “A meniscal tear can lead to knee OA, but knee OA can also lead to a meniscal tear.” Both articular cartilage (AC) (Silvast et al., 2009; Marchiori et al., 2019; Ebrahimi et al., 2020) and menisci (Fithian et al., 1990; Fox et al., 2012; Son et al., 2013; Danso et al., 2017; Travascio et al., 2020a, b; Warnecke et al., 2020; Morejon et al., 2021) are highly anisotropic and inhomogeneous tissues that exhibit strong structure–function relationships that change during the course of OA degeneration. It is well accepted that biomechanical factors like altered joint loading caused by obesity and joint malalignment, trauma, or instability contribute substantially to the initiation and progression of knee joint OA (Hochberg et al., 1995; Jackson et al., 2004; Lohmander et al., 2007; Englund, 2010; Guilak, 2011; Willinger et al., 2019). In the initiation phase, the articulating surfaces already experience structural changes (Andriacchi et al., 2004; Loeser et al., 2012), for example, softening of the AC (Hosseini et al., 2013), while the meniscus loses its elasticity (Fox et al., 2012; Tsujii et al., 2017). Osteoarthritic AC exhibits a decrease in the tensile modulus of up to 90% compared with healthy samples (Akizuki et al., 1986; Setton et al., 1999; Temple-Wong et al., 2009), while Kwok et al. (2014), who examined healthy and degenerated human menisci using nanoindentation, found that the Young’s modulus increased with progressing meniscal degeneration. These findings were underlined by a more recent study utilizing shear wave elastography (Park et al., 2020). In summary, it can be stated that these converse tissue degeneration effects might result in an excessive abrasion of the cartilage tissue accompanied by meniscal tissue calcification, which may finally accelerate knee joint OA progression.

Despite several authors having investigated the biomechanical properties of isolated meniscal and tibial and femoral cartilage specimens utilizing indentation mapping (Sim et al., 2014, 2017; Hadjab et al., 2018; Pflieger et al., 2019; Seidenstuecker et al., 2019), a combined complementary characterization of the articulating partners within the knee joint remains lacking. Consequently, an exact understanding of the interplay between these tissues in relation to the degenerative process is of considerable interest. Therefore, the aim of the present study was to characterize the biomechanical properties of mildly and severely degenerated human knee joints, including their menisci and tibial and femoral AC. Specifically, we wanted to explore the question of whether the degeneration of one of the structures might be the cause of the degenerative processes of the other structure(s), as hypothesized by Englund et al. (2009).

## Materials and Methods

### Study Design

A spatial biomechanical mapping of the articulating knee joint surfaces of 12 mildly and 12 severely degenerated knee joints was assessed using a multiaxial mechanical testing machine. To do so, indentation stress relaxation tests were combined with thickness measurements at the lateral and medial menisci and the AC of the tibial plateau and femoral condyles to finally calculate the instantaneous modulus (IM), the modulus after a relaxation time of 20 s (E_t20_), the maximum applied load (P_max_), and the relaxation percentage over the maximum stress (Δσ_relax_). Additionally, we measured the water content of the tissues and correlated it to the biomechanical data. Non-parametric statistical analyses and correlation analyses were performed to interpret the results.

### Specimen Preparation

**FIGURE 1:**
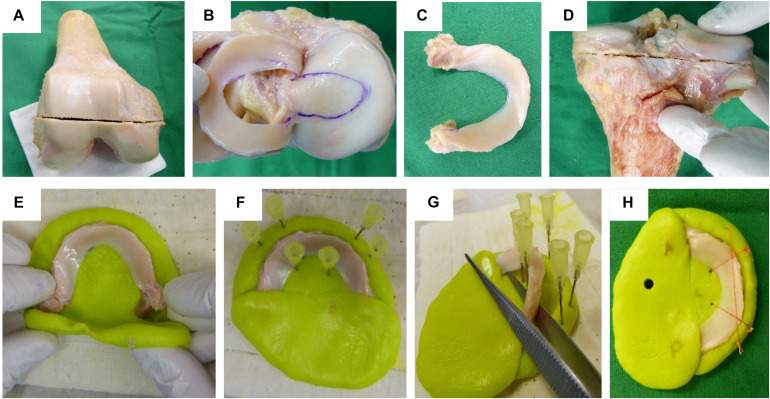
Human cartilage and meniscus sample preparation: **(A)** Horizontal plane cut at the distal femur. **(B)** Highlighting the cartilage-to-cartilage contact area at the tibial plateau using a permanent tissue marker. **(C)** Separated lateral meniscus with its intact anterior and posterior root attachments. **(D)** Horizontal plane cut at the proximal tibia. Meniscus cast preparation steps: **(E)** The meniscus was initially embedded at a time point when the polymethylmethacrylate (PMMA) had a rubber-like texture. **(F)** Insertion of six needles to allow for a later positioning of security sutures. **(G)** During the exothermic hardening reaction of the PMMA, the meniscus body was temporarily removed. **(H)** Final meniscus sample including interlinked security sutures and anterior (A) and posterior (P) cast markings.

Following IRB approval (no. 70/16, Ulm University, Germany), 24 human knee joints were obtained from an official tissue bank (Science Care Inc., Phoenix, AZ, United States). On the basis of Kellgren–Lawrence (KL) grading (Kellgren and Lawrence, 1957) by three independent orthopedic surgeons, the joints were equally assigned to a mild (KL 1–2) and a severe (KL 3–4) degeneration group (Table 1). Prior to dissection, the joints were thawed at room temperature and the skin and soft tissues were removed. Following separation of the femur from the tibial plateau with the menisci remaining attached, the distal part of the femur was cut through a horizontal plane with a band saw to permit mounting on a sample holder (Figure 1A), which was then installed in a testing chamber filled with phosphate-buffered saline (PBS). Then, the menisci were additionally graded using the macroscopic Pauli scoring (Pauli et al., 2011) (Table 1). Subsequently, the meniscus-uncovered cartilage-to-cartilage contact (CtC) area on the lateral and medial compartments of the tibial plateau was marked using a tissue marker (Figure 1B). Following careful detachment of the coronary menisco-tibial ligaments and the connection to the medial collateral ligament of the medial meniscus, both menisci were chiseled out at their anterior and posterior root attachments (Figure 1C). The remaining tibial plateau was cut at a distance of 15 mm parallel to the joint line using a band saw (Figure 1D). Throughout the preparation process, the specimens were kept moist with PBS. During pretests, we recognized a significant circumferential deflection of the menisci when applying normal loading to their femoral surface. Therefore, each meniscus was embedded at its bony attachments by means of a customized polymethylmethacrylate (PMMA; Technovit 3040, Heraeus-Kulzer GmbH, Hanau, Germany) cast, which confined the meniscus along its circumference (Figures 1E,F). During the exothermic setting of the PMMA, special care was taken to avoid any heat-related impact on the menisci (Figure 1G). Furthermore, a trapezoidal interlinked suture material (Figure 1H) secured the menisci in the axial direction and prevented them from a lift-off during the thickness measurements. After the mechanical mapping and thickness measurements were completed, cylindrical samples were extracted from the center of each anatomical region of the menisci [anterior horn (AH), pars intermedia (PI), and posterior horn (PH)] using a biopsy punch (Ø = 4.6 mm, GlaxoSmithKline GmbH, Munich, Germany) (Martin Seitz et al., 2013; Warnecke et al., 2020). The AC samples of the tibial plateau were extracted by means of a trephine drill (Ø = 5.0 mm) at their meniscus corresponding subregions, covered by the meniscus (AH, PI, and PH) and at the CtC area. In a further preparation step, the cartilage layer was separated from the subchondral bone using a previously introduced cutting device (Martin Seitz et al., 2013). Immediately after the preparation process, the wet weight of both the menisci and AC samples were determined using a precision scale (AC120S, Sartorius AG, Göttingen, Germany). All samples were stored in special containers and lyophilized (Lyovac GT2, Finn-Aqua Santasalo-Sohlberg GmbH, Cologne, Germany), dry weighed, and the respective water content was determined.

**TABLE 1:** Demographic data of the mild (upper part) and severe (lower part) degeneration groups.

No.	Side	Gender	Age (years)	Height (cm)	Weight (kg)	BMI (kg/m^2^)	KL score	Pauli score: MM/LM
1	Left	Male	24	196	84	22.1	2	1-1-1/1-1-1
2	Left	Female	53	170	76	26.3	1	1-2-1/1-2-1
3	Right	Female	53	175	68	22.2	2	1-2-2/1-2-1
4	Left	Female	44	168	55	19.7	2	1-1-1/1-1-1
5	Left	Female	68	175	68	22.2	2	1-1-1/1-2-1
6	Right	Male	54	178	86	27.3	2	1-1-1/1-1-1
7	Right	Male	24	196	84	22.1	2	1-1-1/1-1-1
8	Right	Female	53	170	76	26.3	1	1-1-1/1-1-1
9	Left	Male	54	178	86	27.3	2	1-1-1/1-1-1
10	Right	Male	55	175	59	19.0	2	1-1-1/1-1-1
11	Right	Female	44	168	55	19.7	1	1-1-1/1-1-1
12	Left	Male	55	175	59	19.0	2	1-2-1/1-1-1
Mean ± SD	6/6	6/6	48.4 ± 12.9	177 ± 9.5	71.3 ± 12.3	22.8 ± 3.2		
13	Left	Male	70	180	103	33.5	3	2-3-2/4-4-4 (+C)
14	Right	Male	67	175	89	28.9	4	X-X-X/2-2-2 (+C)
15	Right	Male	82	170	66	22.7	4	2-2-2/3-2-2
16	Right	Male	89	168	78	27.8	3	2-3-2/3-2-2
17	Left	Male	82	170	66	22.7	3	2-3-3/2-3-2
18	Right	Male	98	168	75	26.6	4	X-X-X/4-4-3 (+C)
19	Right	Male	89	173	83	27.7	3	2-3-4/1-1-1
20	Right	Male	86	185	77	22.4	3	3-4-3 (+C)/3-3-3 (+C)
21	Left	Male	89	168	78	27.8	4	2-3-2/2-2-2
22	Left	Male	77	188	103	29.1	3	2-2-2/2-2-2
23	Right	Male	77	188	103	29.1	3	1-3-1/1-2-1
24	Left	Male	73	170	94	32.4	3	2-2-2/2-2-2
Mean ± SD	5/7	12/0	81.6 ± 9.1	175.3 ± 7.9	84.6 ± 13.7	27.6 ± 3.6		

*The mean ± SD values of the respective group are summarized in the respective lower gray-shaded row. Kellgren–Lawrence (KL) scores range from 1 (normal knee joints to doubtful degeneration signs) to 4 (severe degeneration with greatly reduced joint space, subchondral sclerosis, etc.). Macroscopic Pauli scoring for the medial (MM) and lateral (LM) menisci, where the degrees of degeneration between 1 for inconspicuous changes/normal tissue and 4 for the most strongly altered tissue were defined. Furthermore, the meniscus regions (anterior horn–pars intermedia–posterior horn) were considered in the macroscopic Pauli meniscus score, while the three subsequent scores represent the respective meniscus region. In the case where the meniscus showed macroscopically visible calcification, (+C) was added. The grading “X” represents the maximum degeneration state where the meniscus was only piecewise available.*

### Biomechanical Testing

A multiaxial mechanical tester (MACH-1 v500css, Biomomentum Inc., Laval, QC, Canada) was used to biomechanically map the articulating surfaces of the menisci, tibial plateau, and femoral condyles of each of the 24 donor knees. Firstly, the built-in camera registration system was used to define repeatable patterns of the measurement points for each surface: the lateral and medial menisci were subdivided into their anatomical subregions (AH, PI, and PH); the tibial plateau was divided into a lateral and a medial CtC area and into the areas that were covered by the corresponding parts of the menisci (AH, PI, and PH). Finally, the distal femur was divided into its lateral and medial condyles. While the AC structures allowed for repeatable mapping grid patterns (tibial plateau compartment: n = 7 × 4; femoral condyles: n = 3 × 6; see Supplementary Material) with similar distances between each measurement point, the lateral and medial menisci received individual grid patterns for the biomechanical mapping. This was necessary because each meniscus was different in its anatomical appearance and degeneration state. In some cases, additional points were manually added. However, a minimum of four measurement points was defined for each meniscal subregion (AH, PI, and PH). An established algorithm (Sim et al., 2017) was applied to create the biomechanical mapping of the respective surfaces: firstly, the surface angle of the structure to be examined is determined. Secondly, according to this surface angle, a normal force is generated by correspondingly moving the three axes of the materials testing machine in a highly accurate coordinated manner. In doing so, a non-destructive indentation test (Table 2) utilizing a spherical indenter (diameter = 2 mm) was conducted to determine the relaxation behavior of each structure. Subsequently, the indenter was replaced with a 26-G 3/8-in. Precision-Glide intradermal bevel needle (BD, Franklin Lakes, NJ, United States) and inserted into the tissue at a test velocity of 0.5 mm/s and a stop criterion of 8 N while recording the force and indentation depth. Once the needle penetrated the tissue surface, the force started to increase. Reaching the subchondral bone or the underlying PMMA cast of the meniscus, respectively, the force response showed a sudden steep increase until the stop criterion was attained. The thickness (h) of the cartilage and menisci was then determined as the needle penetration depth between the point of first force increase and the sudden steep force increase (Jurvelin et al., 1995). Finally, this value was corrected for the surface angulation at the tested position by the cosine of the previously determined angle during the normal indentation procedure (Sim et al., 2017). On the basis of a mathematical least squares fitting of an elastic model in indentation (Hayes et al., 1972) on the experimental data, the IM at each measurement point was determined using the following equation:

**TABLE 2:** Setup data to perform the biomechanical mapping of the cartilaginous (femur and tibia) and meniscus surfaces.

	Contact velocity (mm/s)	Contact criteria (N)	Scanning grid (mm)	Indentation amplitude (mm)	Indentation velocity (mm/s)	Relaxation time (s)
Cartilage	0.5	0.102	0.5	0.2	0.2	20
Meniscus	0.5	0.102	0.5	0.5	0.5	20

$$\mathrm{IM}=\frac{P}{H}\cdot \frac{1-\nu^{2}}{2\,a\,\kappa \,\left(\frac{a}{h_{i}},\nu \right)}$$

where *P* is the applied load in newton, *H* the indentation depth in millimeters ν the Poisson’s ratio, the radius of the contact region in millimeters *h*_*i*_ the thickness at each position in millimeters, and κ is a correction factor that is dependent on *a*/*h* and ν.

In accordance with Seidenstuecker et al. (2019), we identified the *P*_max_, which equals the force registered by the load cell after reaching the AC and the meniscus-specific indentation amplitude. The initial viscous response of the tissues was examined by the determination of the *E*_t20_:

$$E_{\text{t}\,20}=\frac{\sigma_{\text{t}\,20}}{\varepsilon_{i}}=\frac{P_{\text{t}\,20}/A_{\text{sseg}}}{H/h_{i}}$$

where σ_t20_ is the stress in megapascal and ε*_*i*_* is the strain in millimeters per millimeter. *P*_t20_ is the load in *N* after the relaxation time of 20 s and *A*_*sseg*_ is the resultant area of the spherical indentation segment after reaching the according indentation depth (*H*) in square millimeters. The Δσ_relax_ in percent (Coluccino et al., 2017) was determined to gain further insight into the viscoelastic response of the biphasic materials:

$$\Delta \,\sigma_{\text{relax}}=\frac{\sigma_{\text{t}\,20}}{\sigma_{\text{max}}}\,\frac{P_{\text{t}\,20}/A_{\text{sseg}}}{P_{\text{max}}/A_{s\,s\,e\,g}}=\frac{P_{\text{t}\,20}}{P_{\text{max}}}\cdot 100\%$$

where σ_max_ is the maximum stress in megapascal.

### Statistical Analysis

On the basis of the results of a similar study investigating IM differences between healthy and degenerated tibial cartilage (Seidenstuecker et al., 2019), an *a priori* sample size calculation [G^∗^Power 3.1 (Faul et al., 2007): α = 0.05, Power (1 − β) = 0.95, effect size (dz) = 4.71, *n* = 6] was performed to ensure sufficient statistical power of the study. Because no data exist on IM measurements on meniscus samples, the medical epidemiology and statistics department recommended increasing the sample size to *n* = 12 for both groups, resulting in a total sample size of *n* = 24. Gaussian distribution of the data was tested using the Shapiro–Wilk test, resulting in non-normally distributed data. Non-parametric statistical analyses were performed using a statistical software package (SPSS v24, IBM Corp., Armonk, NY, United States). A *p* < 0.05 was considered statistically significant, while *p* value Bonferroni correction was applied, where necessary. Spearman’s rank order correlation coefficients (*r*_s_) were calculated for the *h*, IM, *E*_t20_, *P*_max_, Δσ_relax_, and water content measurements of the mildly and severely degenerated articulating partners: menisci vs. tibial plateau AC, menisci vs. distal femoral AC, and tibial plateau AC vs. distal femoral AC. For detailed analysis, the articulating partners were separated into their lateral and medial compartments to detect possible regional correlations. Ancillary, degeneration-specific *r*_s_ were calculated for the biomechanical parameters (IM, *P*_max_, *E*_t20_, and Δσ_relax_) and the water content of the articulating partners.

## Results

### Menisci

The thickness of the lateral menisci ranged between 1.4 and 8.1 mm and that of the medial menisci between 1.6 and 7.0 mm (Table 3). Meniscal thickness of the different anatomical regions was neither different for the mildly degenerated menisci (Friedman test: *p* > 0.56) nor for the severely degenerated menisci (*p* > 0.34). By tendency, the severely degenerated lateral and medial menisci were 23% and 16% thicker, respectively, compared to their mildly degenerated counterparts. Mann–Whitney *U* tests revealed an 88% greater meniscal thickness at the PH of the severely degenerated lateral menisci (*p* < 0.01) and a 69% thicker PI at the severely degenerated medial menisci (*p* = 0.03) compared to their mildly degenerated counterparts.

**TABLE 3:** Thickness measurement results (minimum, median, and maximum values) of the cartilaginous (femur and tibia) and meniscus (AH, PI, PH, and CtC area) surface localizations.

		Lateral				Medial			
		AH	PI	PH	CtC	AH	PI	PP	CtC
**Mild degeneration**									
Meniscus	Min	2.7	1.4	1.8		1.6	1.8	2.7	
	Median	3.4	3.0	3.4*		3.2	2.4*	3.8	
	Max	7.0	8.1	5.3		7.0	4.8	4.9	
Tibial plateau	Min	1.7	1.5	1.7	2.7	1.4	1.4	1.7	1.9
	Median	2.0*	2.1*	2.4	3.4	1.8	2.0	2.0	2.3
	Max	3.4	2.9	3.4	4.3	2.5	2.9	3.0	3.0
Distal femur	Min	1.7				1.4			
	Median	2.1				2.7			
	Max	3.2				4.0			
**Severe degeneration**									
Meniscus	Min	1.9	2.2	3.1		2.8	2.3	2.2	
	Median	4.2	3.2	4.7*		3.7	3.7*	3.7	
	Max	6.9	5.6	6.0		6.1	5.1	6.1	
Tibial plateau	Min	1.4	0.9	2.1	2.5	1.1	0.8	1.8	1.3
	Median	3.0*	2.9*	2.5	3.5	2.1	2.1	2.3	2.2
	Max	3.7	3.8	3.7	4.7	4.3	3.0	2.8	3.5
Distal femur	Min	1.6				1.6			
	Median	2.2				2.4			
	Max	3.4				3.2			

*Non-parametric statistical analyses: *n* = 12.*

AH, anterior horn; PI, pars intermedia; PH, posterior horn; CtC, cartilage-to-cartilage area.

*p < 0.05.

**FIGURE 2:**
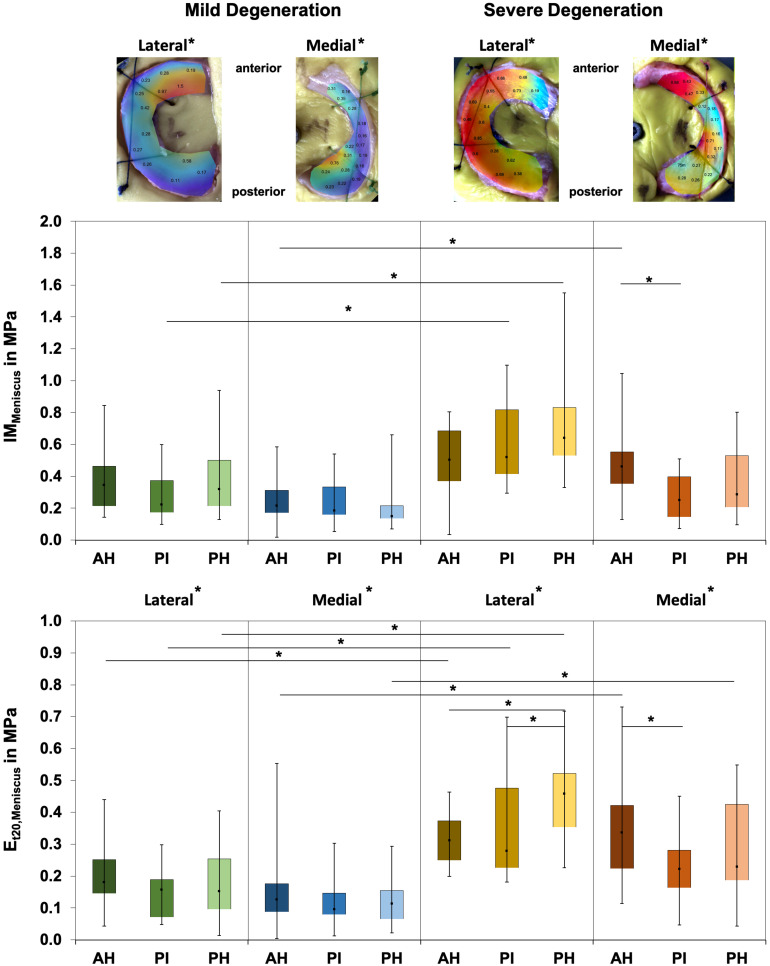
Representative biomechanical mappings of the instantaneous modulus (IM) measurements of mildly and severely degenerated menisci, with all values given in megapascal. *Middle row*: Box plots (minimum, maximum, median, and 25th and 75th percentiles) of the lateral and medial IM_Meniscus_ values in megapascal. *Lower row*: Box plots (minimum, maximum, median, and 25th and 75th percentiles) of the lateral and medial *E*_t20_ values in megapascal. Subdivided anatomical regions are: AH, anterior horn; PI, pars intermedia; PH, posterior horn. Non-parametric statistical analyses: *n* = 12; *p < 0.05. For reasons of readability, we marked significant differences between the mild and severe degeneration of the medial and lateral sides *above the representative biomechanical mappings*.

Friedman testing revealed significant differences for the IM, which ranged between 0.1 and 1.58 MPa, only at the severely degenerated medial menisci (*p* = 0.01; Figure 2) and for the localizations AH vs. PI (*p* = 0.01). Lateral menisci had a higher IM than medial menisci for both the mild (*p* = 0.01) and severe (*p* = 0.01) degeneration groups. Compared to the corresponding mildly degenerated menisci, the severely degenerated lateral and medial menisci displayed a 64% higher (Mann–Whitney *U* test: *p* = 0.01) and a 54% higher (*p* = 0.02) IM, respectively. In detail, the severe lateral PI (Mann–Whitney *U* test: *p* < 0.01) and lateral PH (*p* < 0.01) indicated 100% and 91%, respectively, higher IM values compared to their mildly degenerated lateral PI and PH counterparts. Regarding the anatomical region degeneration comparison of the medial menisci, only the AH of the severely degenerated menisci indicated a statistically relevant 66% higher IM value (*p* = 0.03) compared to the mildly degenerated menisci. The *E*_t20_ of the mildly degenerated menisci ranged between 0.01 and 0.55 MPa (Figure 2) and those of the severely degenerated menisci between 0.04 and 0.73 MPa. While Friedman testing revealed no differences regarding the anatomical subregions of the mildly degenerated menisci, the severely degenerated menisci indicated differences both at the lateral (*p* = 0.04) and medial (*p* < 0.01) menisci. Consecutive Wilcoxon testing showed a statistically higher *E*_t20_ of the lateral PH vs. AH (*p* = 0.03) and PH vs. PI (*p* = 0.01), while for the severely degenerated medial menisci, the *E*_t20_ of the AH was higher compared to the PI (*p* < 0.01). Compared to the mildly degenerated menisci, the *E*_t20_ values of the severely degenerated menisci were 127% (Mann–Whitney *U* test: *p* < 0.01) and 75% (*p* < 0.01) higher on the lateral and medial sides, respectively. All anatomical subregions of the severely degenerated menisci indicated significantly higher *E*_t20_ values (Mann–Whitney *U* test: AH: 91%, *p* < 0.01; PI: 137%, *p* < 0.01; PH: 136%, *p* < 0.01) compared to their mildly degenerated counterparts. The severely degenerated AH indexed a 78% higher (*p* = 0.02) and the PH an 84% higher (*p* = 0.01) *E*_t20_ compared to their mildly degenerated counterparts.

The *P*_max_ values of the mildly degenerated menisci ranged between 0.01 and 1.02 N without subregional differences (Friedman test: *p* > 0.17; Table 4). The severely degenerated medial menisci displayed subregional differences (*p* = 0.02), with a 114% higher *P*_max_ only for the comparison between the AH and PI (Wilcoxon test: *p* = 0.02). Both lateral and medial severely degenerated menisci displayed a 47% (*p* < 0.01) and 53% (*p* < 0.01), respectively, higher *P*_max_ than their mildly degenerated comparators. In detail, compared to the mildly degenerated menisci, the severely degenerated lateral PI (Mann–Whitney *U* test: *p* < 0.01) and medial AH (*p* < 0.01) indicated 88% and 105% higher *P*_max_ values, respectively.

**TABLE 4:** Maximum applied load, *P*_max_ (minimum, median, and maximum values), of the cartilaginous (femur and tibia) and meniscus (AH, PI, PH, and CtC area) surface localizations.

		Lateral				Medial			
		AH	PI	PH	CtC	AH	PI	PP	CtC
**Mild degeneration**									
Meniscus	Min	0.03	0.03	0.03		0.01	0.06	0.04	
	Median	0.40	0.33*	0.41		0.21*	0.24	0.18	
	Max	1.02	0.72	0.91		0.49	0.59	0.79	
Tibial plateau*	Min	0.10	0.84	0.04	0.04	0.11	0.31	0.72	0.15
	Median	2.35*	3.09*	0.52*	0.45	3.15*	4.15*	2.63*	1.46
	Max	4.13	5.07	1.52	1.03	6.24	6.78	4.31	3.86
Distal femur	Min	0.6				0.5			
	Median	2.3				2.9			
	Max	5.7				5.2			
**Severe degeneration**									
Meniscus	Min	0.18	0.26	0.37		0.13	0.05	0.11	
	Median	0.57	0.60*	0.50		0.58*	0.27*	0.33	
	Max	0.94	0.85	1.12		0.92	0.63	0.86	
Tibial plateau*	Min	0.61	0.85	0.11	0.06	0.22	0.94	1.29	0.12
	Median	1.20	2.35	0.36	0.10	1.18*	1.50*	2.03	0.64*
	Max	3.75	9.24	3.24	1.53	2.39	3.74	4.39	1.65
Distal femur	Min	0.2				0.8			
	Median	2.9*				2.2*			
	Max	4.2				3.0			

*Non-parametric statistical analyses: *n* = 12.*

AH, anterior horn; PI, pars intermedia; PH, posterior horn; CtC, cartilage-to-cartilage area.

*p < 0.05.

**FIGURE 3:**
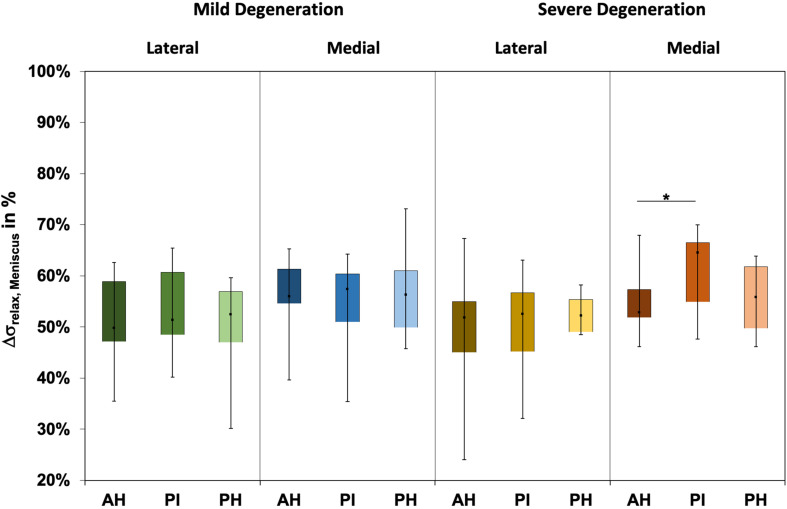
Box plots (minimum, maximum, median, and 25th and 75th percentiles) of the relaxation percentages over the maximum stress (Δσ_relax_) of the mildly and severely degenerated menisci. Subdivided anatomical regions are: AH, anterior horn; PI, pars intermedia; PH, posterior horn. Non-parametric statistical analyses: *n* = 12; *p < 0.05.

The Δσ_relax_ (Figure 3) ranged between 24% and 73% and indicated subregional differences only for the severely degenerated medial menisci (Friedman test: *p* = 0.01) between the AH and PI (Wilcoxon test: *p* = 0.03). The Δσ_relax_ was identical for the mildly and severely degenerated menisci (Mann–Whitney *U* test: *p* > 0.71).

**FIGURE 4:**
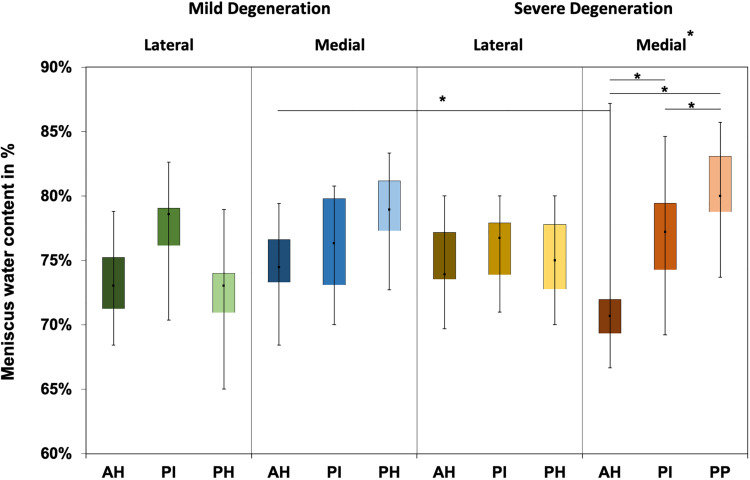
Water content value box plots (minimum, maximum, median, and 25th and 75th percentiles) in percent of the mildly and severely degenerated menisci. Subdivided anatomical regions are: AH, anterior horn; PI, pars intermedia; PH, posterior horn. Non-parametric statistical analyses: *n* = 12; *p < 0.05.

The water content of the menisci ranged between 65% and 87% (Figure 4), while only the severely degenerated medial menisci indicated subregional differences (Friedman test: *p* < 0.01). In detail, the AH indicated significantly less water content compared to the corresponding PI (−8%, *p* = 0.04) and PH (−12%, *p* = 0.01), and the PI contained more water (4%, *p* = 0.01) than the PH. By tendency, the severely degenerated menisci showed a higher water content both for the lateral (∼2%) and medial (∼1%) compared to the mildly degenerated menisci.

### Tibial Cartilage

The thickness of the AC of the tibial plateau ranged between 0.8 mm, measured at the PI at the medial compartment of the severely degenerated joints, and 4.7 mm, measured at the CtC area at the lateral compartment of the severely degenerated joints (Table 3). Friedman testing revealed a statistical difference for the thickness measurements between both the medial and lateral subregions (AH, PI, and PH) of the mildly degenerated knees (*p* < 0.01). Regarding the severely degenerated knees, only the lateral compartment indicated subregional differences (Friedman test: *p* < 0.01). In general, the cartilage at the CtC area was always thicker compared to the meniscus-covered cartilage. Severely degenerated tibial cartilage was, on average, 29% (Mann–Whitney *U* test: *p* < 0.01) thicker compared to the mildly degenerated tibial cartilage. Detailed analyses revealed that the severely degenerated menisci were thicker at the lateral AH (73%, *p* = 0.02) and lateral PI (75%, *p* = 0.02) compared to their mildly degenerated counterparts.

**FIGURE 5:**
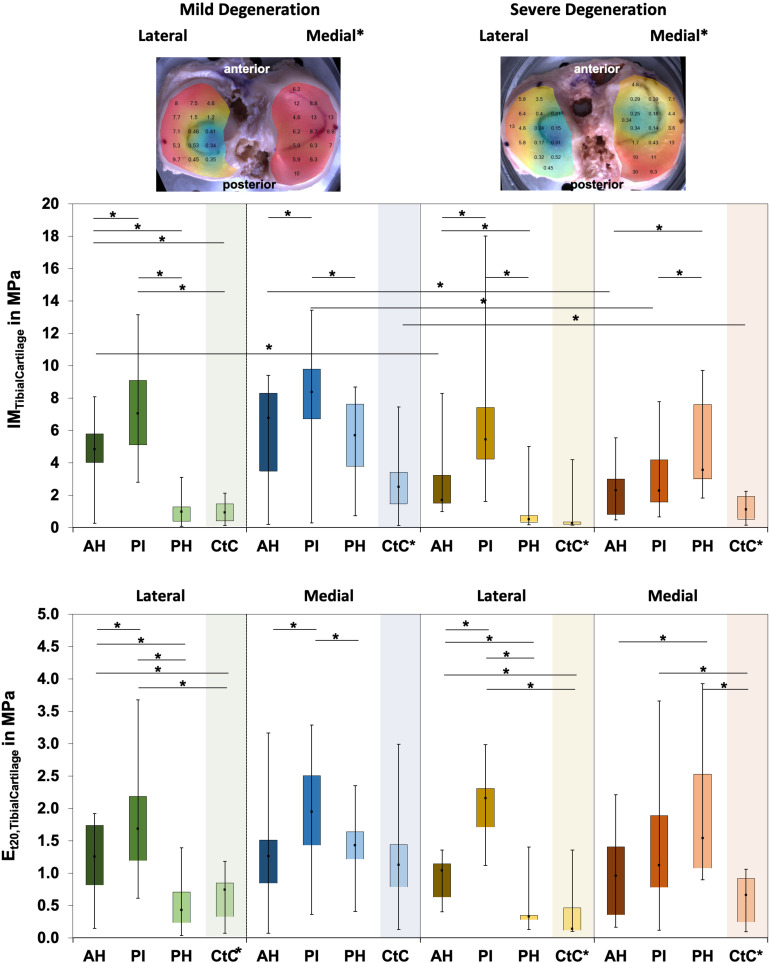
Representative biomechanical mappings of the instantaneous modulus (IM) measurements of the mildly and severely degenerated tibial plateau articular cartilage (AC), with all values given in megapascal. *Middle row*: Box plots (minimum, maximum, median, and 25th and 75th percentiles) of the lateral and medial IM_*TibialCartilage*_ values in megapascal. *Lower row*: Box plots (minimum, maximum, median, and 25th and 75th percentiles) of the lateral and medial *E*_t20_ values in megapascal. Subdivided anatomical regions are: *AH*, anterior horn; *PI*, pars intermedia; *PH*, posterior horn; *CtC*, cartilage-to-cartilage contact area. Non-parametric statistical analyses: *n* = 12; *p < 0.05. For reasons of readability, we marked significant differences between the mild and severe degeneration of the medial and lateral sides *above the representative biomechanical mappings* and also between the CtC and other anatomical compartments only *at the legend of the category axis* (e.g., CtC*).

The IM values for tibial AC covered by the menisci were localization-dependent (Friedman test: *p* < 0.02; Figure 5) and ranged between 0.1 and 17.6 MPa. In detail, the lateral compartment of the mildly degenerated knees displayed differences for all localizations (*p* < 0.01), while the medial compartment showed differences at the AH vs. PI (*p* < 0.01) and the PI vs. PH (*p* = 0.01). For the severely degenerated joints, the IM was different at all localizations on the lateral compartment (*p* < 0.01) and on the medial compartment at the AH vs. PH (*p* < 0.01) and the PI vs. PH (*p* = 0.01). Comparing the areas covered by the menisci with the uncovered CtC area showed lower IM values for the CtC area (*p* < 0.03), except for the lateral PH of the mildly degenerated joints (*p* = 0.53). At the mildly degenerated joints, the IM was higher for the medial than for the lateral side (Mann–Whitney *U* test: *p* < 0.01), while for the severely degenerated joints the IM was similar for both compartments. Degeneration had no influence on the lateral cartilage IM (Mann–Whitney *U*: *p* = 0.17), while the medial compartment cartilage of the mildly degenerated joints had higher IM values (*p* < 0.01) than those of the severely degenerated joints. Mann–Whitney *U* tests indicated that the lateral AC compartments displayed a significantly higher IM (68%, *p* = 0.03) for the mildly degenerated AH subregion compared to its severely degenerated counterpart. On the medial side, the mildly degenerated AC indicated higher IM values at the subregions of the AH (84%, *p* = 0.02) and PI (94%, *p* < 0.01) and at the meniscus-uncovered CtC region (65%, *p* = 0.04) compared to their severely degenerated counterparts. Comparable to the IM results, the *E*_t20_ values for the meniscus-covered tibial AC were also different for the subregions (Friedman test: *p* < 0.02) and ranged between 0.04 and 3.93 MPa (Figure 5). The lateral mildly (Wilcoxon test: *p* < 0.03) and severely (*p* < 0.01) degenerated tibial AC showed differences between all anatomical localizations, with the highest values at the PI subregion. The mildly degenerated medial tibial AC indicated differences between the *E*_t20_ values of the AH vs. PI (*p* < 0.01) and the PI vs. PH (*p* < 0.01), also with the highest values at the PI subregion. The severely degenerated medial tibial AC indicated statistically higher *E*_t20_ values only for the PH vs. AH (60%, *p* = 0.04). Comparisons between the meniscus-covered and uncovered AC regions indicated differences between the mildly degenerated lateral AH (*p* < 0.01) and PI (*p* < 0.01), with significantly higher values for the meniscus-covered subregions compared to the CtC region. Severely degenerated meniscus-covered lateral AC had a 7.6 times higher *E*_t20_ (*p* < 0.01) at the AH and 16 times higher (*p* < 0.01) at the PI compared to the CtC region. Both the PI (*p* = 0.04) and PH (*p* < 0.01) of the severely degenerated medial menisci had significantly higher values compared to the meniscus-uncovered CtC region. There was neither for the lateral nor for the medial tibial AC compartments a difference of the *E*_t20_ values (Mann–Whitney *U* test: *p* > 0.07) between the mild and severe degeneration states.

The *P*_max_ of the degenerated tibial AC ranged between 0.04 and 9.24 N (Table 4). The *P*_max_ showed subregional dependencies for the lateral and medial compartments and for both degeneration conditions (Friedman test: *p* < 0.03). In detail, the *P*_max_ values of the mildly and severely degenerated lateral tibial AC subregions were different (Wilcoxon test: *p* < 0.01) and indicated a descending *P*_max_ from the PI (3.11 and 2.35 N, respectively) to the AH (72% and 51%, respectively) to the PH (21% and 15%, respectively). The mildly degenerated medial AC also showed the highest *P*_max_ value at the PI, which was statistically higher, compared to the AH (31%, *p* = 0.02) and the PH (46%, *p* < 0.01). The severely degenerated tibial medial AC was only different for the comparison between the AH and PH (*p* < 0.01). The meniscus-covered tibial AC indicated statistically higher *P*_max_ values (Wilcoxon test: *p* < 0.04) compared to the respective CtC regions, with the exception of the mildly degenerated lateral AC at the PH subregion (*p* = 0.31). Progressing degeneration significantly decreased the *P*_max_ (−34%; Mann–Whitney *U* test: *p* < 0.01) of the medial tibial AC, while the lateral side remained unaffected (*p* = 0.16). In detail, the *P*_max_ at the medial tibial AC decreased with progressing degeneration at the AH (−43%; Mann–Whitney *U* test: *p* = 0.03), PI (−50%, *p* < 0.01), and the meniscus-uncovered CtC region (−39%, *p* = 0.05).

**FIGURE 6:**
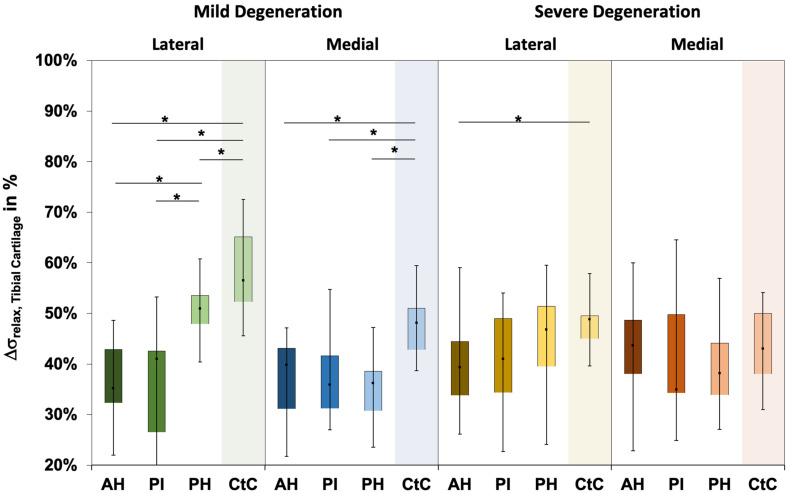
Box plots (minimum, maximum, median, and 25th and 75th percentiles) of the relaxation percentages over the maximum stress (Δσ_relax_) of the mildly and severely degenerated tibial plateau articular cartilage (AC). Subdivided anatomical regions are: AH, anterior horn; PI, pars intermedia; PH, posterior horn; CtC, cartilage-to-cartilage contact area. Non-parametric statistical analyses: *n* = 12; *p < 0.05.

The Δσ_relax_ ranged between 15 and 73% for the mildly and between 23% and 65% for the severely degenerated tibial AC (Figure 6). Friedman testing indicated subregional differences for the mildly degenerated lateral tibial AC (*p* < 0.01) between the AH and PH (Wilcoxon test: *p* < 0.01) and the PI and PH (*p* < 0.01). Mildly degenerated joints indicated significantly higher Δσ_relax_ values of the uncovered CtC area compared to all lateral (Wilcoxon test: *p* < 0.01) and medial meniscus-covered subregions (*p* < 0.01). Regarding the severely degenerated tibial AC, only the lateral AH and the lateral CtC area showed significant differences (*p* = 0.03). There was no statistical difference for the Δσ_relax_ values between the mildly and severely degenerated tibial AC (Mann–Whitney *U* test: *p* > 0.05).

**FIGURE 7:**
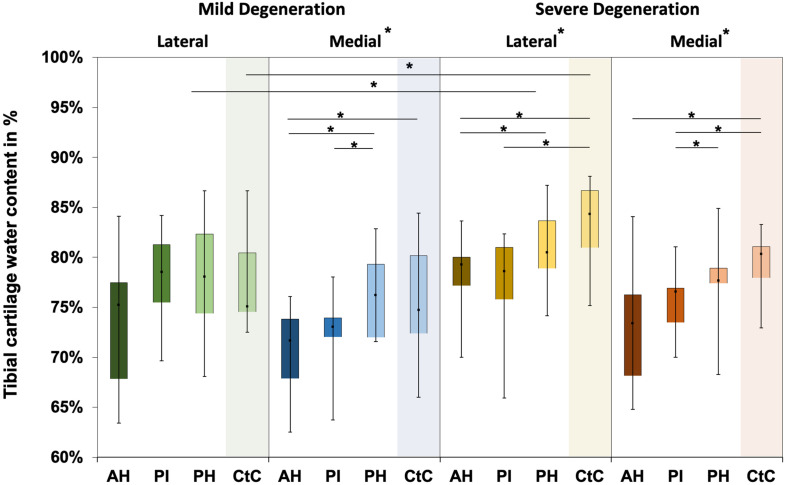
Water content in percent of the mildly and severely degenerated tibial plateau articular cartilage (AC). Box plots (minimum, maximum, median, and 25th and 75th percentiles) of the tibial cartilage water content values in percent. Subdivided anatomical regions are: AH, anterior horn; PI, pars intermedia; PH, posterior horn; CtC, cartilage-to-cartilage contact area. Non-parametric statistical analyses: *n* = 12; *p < 0.05.

Water content ranged between 63% and 87% for the mildly degenerated and between 65% and 88% for the severely degenerated tibial AC (Figure 7). With the exception of the mildly degenerated lateral tibial AC, Friedman testing revealed subregional water content differences at the mildly degenerated medial (*p* < 0.01) and both severely degenerated (lateral: *p* = 0.04; medial: *p* = 0.03) tibial AC compartments. In general, the AH indicated the lowest water content of all the subregions. Wilcoxon testing indicated an 11% higher water content for the PH vs. AH (*p* < 0.01), 10% higher for the CtC vs. AH (*p* = 0.02), and 6% higher for the PH vs. PI (*p* = 0.05) at the mildly degenerated medial tibial AC. Detailed comparisons of the severely degenerated lateral AC indicated a 6% higher water content for the comparison of the PH vs. AH (*p* = 0.02), 11% higher water content for the CtC area AH (*p* = 0.01), and 10% higher water content for the CtC area compared to the PI (*p* = 0.01). The severely degenerated medial PI subregion indicated 7% less water content compared to PH (*p* < 0.01) and 11% less compared to the CtC area (*p* = 0.02). Moreover, the CtC area indicated 12% more (*p* = 0.02) water content than the AH subregion. The severely degenerated tibial AC contained significantly more water than the mildly degenerated tibial AC (lateral: 9%, *p* < 0.01; medial: 3%, *p* = 0.04). Consecutive Mann–Whitney *U* tests revealed a statistically higher water content at the severely degenerated lateral PH (10%, *p* = 0.03) and lateral CtC area (18%, *p* = 0.03) compared to their mildly degenerated counterparts.

### Distal Femur Cartilage

The thickness of the mildly and severely degenerated distal femur cartilage ranged between 1.4 and 4.0 mm and showed similar values (*p* > 0.24) for both the lateral and medial compartments (Table 3).

**FIGURE 8:**
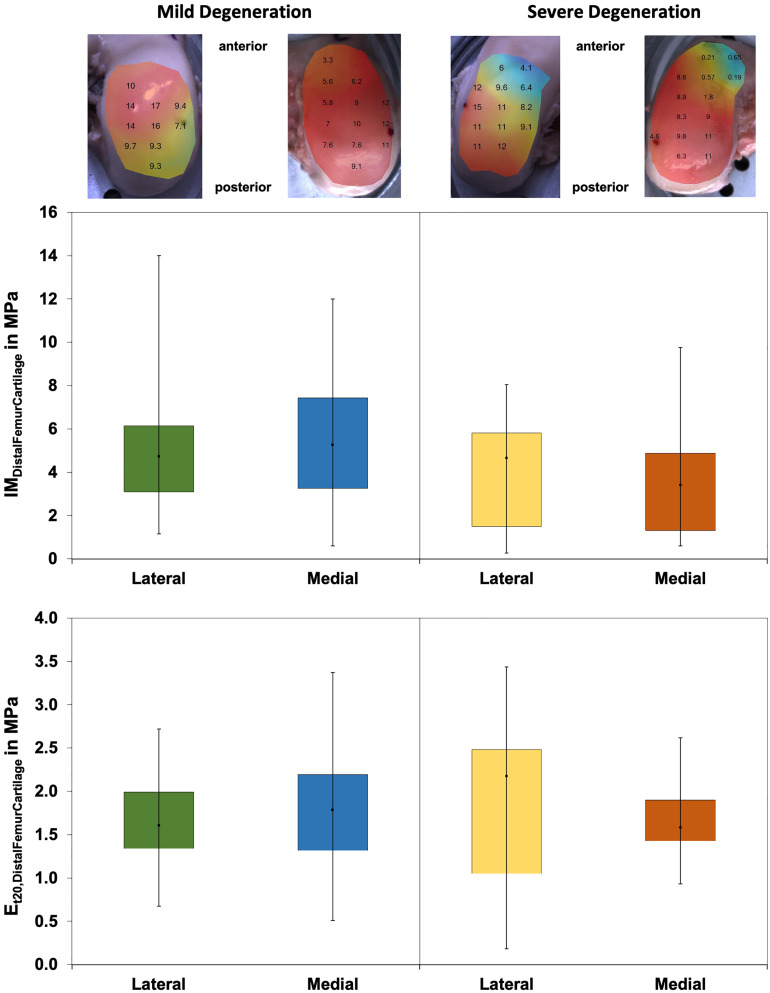
Representative maps of the instantaneous modulus (IM) measurements of the mildly degenerated and severely degenerated distal femora, with all values given in megapascal. *Middle row*: Box plots (minimum, maximum, median, and 25th and 75th percentiles) of the lateral and medial IM_*DistalFemurCartilage*_ values in megapascal. *Lower row*: Box plots (minimum, maximum, median, and 25th and 75th percentiles) of the lateral and medial *E*_t20_ values in megapascal. Non-parametric statistical analyses: *n* = 12.

Wilcoxon testing indicated differences neither between the medial and lateral distal femur cartilage IM values (*p* > 0.58; Figure 8) nor in the *E*_t20_ values (*p* > 0.45). Although the IM values of the severely degenerated medial distal femur cartilage were 35% lower (lateral: 2% lower) compared to its mildly degenerated counterpart, the values were not different (*p* > 0.21). The *E*_t20_ values were also not different (Mann–Whitney *U* test: *p* > 0.57) between the mild and severe degeneration groups.

The *P*_max_ values were different between the severely degenerated lateral and medial distal femoral AC, indicating significantly higher values for the lateral side (Wilcoxon test: 34%, *p* = 0.03; Table 4). Progressing degeneration had no influence on the *P*_max_ of the distal femoral AC (Mann–Whitney *U* test: *p* > 0.18).

**FIGURE 9:**
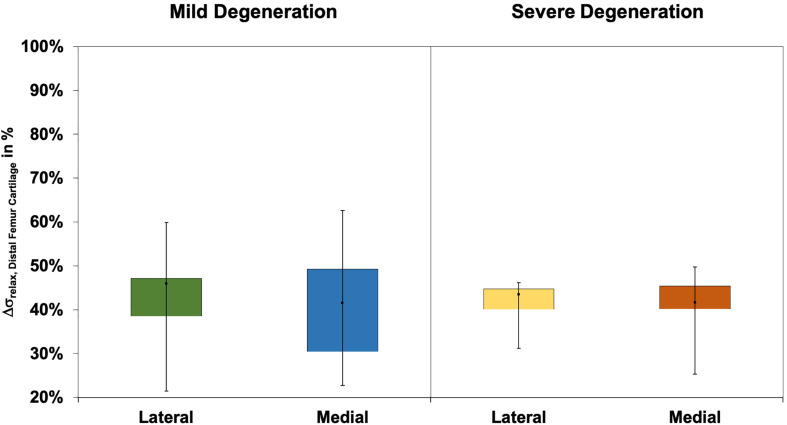
Box plots (minimum, maximum, median, and 25th and 75th percentiles) of the relaxation percentages over the maximum stress (Δσ_relax_) of the mildly and severely degenerated distal femur articular cartilage (AC). Non-parametric statistical analyses: *n* = 12.

The Δσ_relax_ values (Figure 9) of the distal femur AC ranged between 21% and 63% and indicated no difference between the lateral and medial condyles (*p* > 0.39). The degeneration progression also had no influence on the distal femur AC Δσ_relax_ (Mann–Whitney *U* test: *p* > 0.32).

### Correlations

The thickness of the severely degenerated lateral menisci and the corresponding tibial plateau thickness indicated a significant correlation (*r*_s_ = 0.36, *p* = 0.03), whereas the menisci and femur AC thickness did not correlate with each other. The thickness of the severely degenerated lateral tibial CtC and the corresponding severely degenerated lateral femoral AC correlated well (*r*_s_ = 0.86, *p* < 0.01). Both the IM (*p* > 0.12) and *E*_t20_ (*p* > 0.12) values of the mildly and severely degenerated meniscal, tibial, and femoral AC did not correlate with each other. The *P*_max_ values of the meniscus and tibia (*p* > 0.68) as well as the meniscus and femur (*p* > 0.37) did not correlate. The *P*_max_ of the mildly degenerated tibial CtC AC and the mildly degenerated corresponding femoral AC showed a significant correlation (*r*_s_ = 0.62, *p* = 0.03). The Δσ_relax_ of the severely degenerated medial menisci and of the severely degenerated tibial AC indicated a significant negative correlation (*r*_s_ = −0.42, *p* = .04), whereas the menisci and femur, as well as the tibial CtC and femur AC, Δσ_relax_ did not correlate with each other. The water content of the menisci and the according tibial plateau AC did not correlate (*p* > 0.15).

There were significant correlations for both mildly and severely degenerated lateral and medial menisci for the IM vs. *E*_t20_ (*r*_s_ > 0.64, *p* < 0.01), IM vs. *P*_max_ (*r*_s_ > 0.48, *p* < 0.01), IM vs. Δσ_relax_ (*r*_s_ < −0.36, *p* < 0.03), *E*_t20_ vs. *P*_max_ (*r*_s_ > 0.34, *p* < 0.04), and *P*_max_ vs. Δσ_relax_ (*r*_s_ < −0.55, *p* < 0.01). The water content showed only a correlation at the severely degenerated medial menisci for the IM measurements (*r*_s_ = −0.55, *p* < 0.01).

Tibial plateau AC indicated significant Spearman’s correlations for the IM vs. *E*_t20_ (*r*_s_ > 0.79, *p* < 0.01), IM vs. *P*_max_ (*r*_s_ > 0.80, *p* < 0.01), and *E*_t20_ vs. *P*_max_ (*r*_s_ > 0.69, *p* < 0.01), while the *E*_t20_ vs. Δσ_relax_ (*r*_s_ = 0.50, *p* < 0.01) correlated only for the severely degenerated medial tibial plateau AC. Additionally, the mildly and severely degenerated lateral tibial plateau AC indicated correlations for the IM vs. Δσ_relax_ (*r*_s_ < −0.43, *p* < 0.01) and *P*_max_ vs. Δσ_relax_ (*r*_s_ < −0.55, *p* < 0.01). The biomechanical properties of the lateral tibial plateau and the mildly degenerated medial tibial plateau AC did not correlate with the water content. The water content showed a correlation only for the *E*_t20_ (*r*_s_ = 0.45, *p* = 0.03) at the severely degenerated medial tibial AC.

The mildly and severely degenerated CtC area of the lateral and medial tibial plateaus showed significant correlations for the IM vs. *E*_t20_ (*r*_s_ > 0.79, *p* < 0.01), IM vs. *P*_max_ (*r*_s_ > 0.77, *p* < 0.01), and *E*_t20_ vs. *P*_max_ (*r*_s_ > 0.62, *p* < 0.05).

Both mildly and severely degenerated lateral femoral AC indicated significant correlations for the IM vs. *E*_t20_ (*r*_s_ > 0.74, *p* < 0.01), IM vs. *P*_max_ (*r*_s_ > 0.70, *p* < 0.02), and *E*_t20_ vs. *P*_max_ (*r*_s_ > 0.60, *p* < 0.05). The mildly degenerated medial femoral AC correlated for the IM vs. *P*_max_ (*r*_s_ = 0.99, *p* < 0.01), while the severely degenerated medial femoral AC showed significant correlations for the IM vs. Δσ_relax_ (*r*_s_ = −0.66, *p* = 0.04), *E*_t20_ vs. *P*_max_ (*r*_s_ = 0.64, *p* = 0.05), and *P*_max_ vs. Δσ_relax_ (*r*_s_ = −0.79, *p* < 0.01).

The water content did not correlate with the meniscus thickness. Only the mildly degenerated lateral tibial plateau cartilage and the according CtC area showed a positive correlation between the water content and cartilage thickness (*r*_s_ = 0.50, *p* < 0.01, and *r*_s_ = 0.69, *p* < 0.03, respectively).

## Discussion

The most important finding of this study is that, with progressing joint degeneration, there was an increase in the lateral and medial meniscal IM, *E*_t20_, and *P*_max_ values, while the underlying tibial AC indicated an IM and *P*_max_ decrease only at the medial compartment. The stress relaxation percentage Δσ_relax_, which could be interpreted as a measure for the viscous properties of the menisci (Coluccino et al., 2017), was not affected by the degeneration of any of the investigated articulating partners. OA progression had no influence on any of the investigated biomechanical parameters of the distal femoral AC. Furthermore, our water content analyses indicated that, with progressing OA degeneration, the tibial AC water content significantly increased, while the menisci showed only a tendential increase.

Our results suggest that, in severely degenerated joints, the meniscal stiffness increased, whereas the femoral and tibial AC softened. Particularly at the lateral compartment, changes of the biomechanical parameters (IM, *E*_t20_, and *P*_max_) were detectable in the menisci prior to the AC. This could be interpreted as a first sign that OA-related knee joint degeneration first initiates a stiffening of the meniscus surface, followed by a softening of the AC. This could lead to disturbed tibiofemoral contact transmission, where the menisci might play an active role by causing premature cartilage degeneration. In contrast to the severely degenerated joints, the mildly degenerated joints displayed positive correlation values for the IM, where the stiffness for both the meniscus and AC increased. Therefore, it appears that there is a degeneration-dependent pivot point of correlation. Furthermore, we believe that this is the first study that comprehensively investigated the biomechanical changes of the articulating knee joint partners during different degeneration grades. In particular, the preparation technique to allow for the biomechanical mapping of the menisci is described here for the first time.

### Thickness Measurements

Although the needle indentation method is the gold standard for AC thickness measurement (Horbert et al., 2019), to our knowledge, this is the first time that this method has been applied to degenerated menisci. Indeed, the ranges measured in the present study were in accordance with those obtained with *in vivo* magnetic resonance imaging (Erbagci et al., 2004; Bamac et al., 2006; Hunter et al., 2006) and *ex vivo* caliper measurements (Takroni et al., 2016). The AC thickness results of the present study are in agreement with those given in the literature: while in the early degeneration phase the cartilage matrix indicates hypertrophic swelling (Buck et al., 2010), resulting in increased thickness (Hunter et al., 2008; Buck et al., 2010, 2013; Sim et al., 2017), which is also shown in the CtC area of our mildly degenerated joints, and further progression of cartilage degeneration leads to reduced structural integrity (Waldstein et al., 2016). This, combined with increased wear, culminates in substantial cartilage volume loss associated with decreased cartilage thickness (Stefanik et al., 2016; Waldstein et al., 2016; Horbert et al., 2019; Seidenstuecker et al., 2019), which is in accordance with the measurements found particularly at the medial compartments of the severely degenerated knees of the present study. Furthermore, the mild (six females/six males) and severe degeneration (no female/12 males) groups were clearly different in gender distribution. It is known that male knee joints display not only up to 46.6% higher cartilage volumes compared to female knee joints but also a 13.3% higher mean cartilage thickness (Faber et al., 2001). This unequal gender distribution might have further amplified the AC thickness difference between the mild and severe degeneration groups.

### Biomechanical Mapping

We found an increasing IM with progressing meniscal degeneration for both the lateral and medial menisci, ranging from 0.1 to 1.6 MPa. This range is in agreement with the values published for healthy and degenerated human menisci (Moyer et al., 2012, 2013; Kwok et al., 2014; Danso et al., 2015; Fischenich et al., 2015). While Danso et al. (2015) and Fischenich et al. (2015) used a likely similar method to the present study, Kwok et al. (2014) applied atomic force microscopy and Moyer et al. (2012, 2013) used nanoindentation to determine the IM of human meniscal tissue. However, only two of these studies (Kwok et al., 2014; Fischenich et al., 2015) focused on the impact of meniscal degeneration on the IM. Whereas Fischenich et al. (2015) observed an IM decrease with progressing degeneration, Kwok et al. (2014) reported, similar to the results in the present study, an IM increase with progressing degeneration. A possible explanation for the contrary IM trends between the study of Fischenich et al. (2015) and the present study might be the different scoring methods that were principally used to group the specimens. While Fischenich et al. (2015) used the macroscopic Pauli score (Pauli et al., 2011) with detailed subcategories from 1 to 4, we applied the radiographic KL score to assign the specimens initially into two groups, combining the lower and higher KL scores, respectively. Furthermore, the different testing methods might also have an impact on the contrary outcomes. While we analyzed multiple testing points within one anatomical region and extracted the respective median values, the group of Fischenich used only one testing point within each region. However, at the posterior meniscus region, which was the region with the most homogeneous sample distribution with regard to the degeneration state, Fischenich et al. (2015) also reported both an increase in the instantaneous compressive modulus and an increase in the equilibrium compressive modulus with progressing degeneration. Additionally, we confined the specimen along the circumference because we observed during pretests a recognizable radial deflection of the menisci during the normal indentation tests, particularly on the femoral-facing outer meniscal rim. The region-specific differences observed in the current study coincide with the findings from other studies (Moyer et al., 2012; Danso et al., 2017).

In contrast to the increased lateral and medial meniscal IM with progressing degeneration, we found a statistical IM decrease only at the medial tibial plateau AC. Although not significant, there was a softening tendency of both the tibial plateau and femoral condyle surfaces. This trend and the IM values of the femoral condyles measured in the current study (range = 0.5–9 MPa) are in accordance with those reported by Sim et al. (2014, 2017). However, unlike Sim et al. (2017), the IM values of the AC of the femoral condyles did not display significant changes with progressing degeneration, although a trend toward a decrease in the IM with degeneration was observed, particularly on the medial side. This difference could be explained by the different donor groups and the different non-correlating OA grading scores (Down et al., 2011) used in the comparison studies. While Sim et al. *post hoc* graded the asymptomatic articular surfaces of their eight specimens on an adapted macroscopic Committee of the International Cartilage Repair Society (ICRS) score (Mainil-Varlet et al., 2003) and histological Mankin scoring (Mankin et al., 1981), we grouped our 24 knees *a priori* into two different degeneration groups, which was based on KL and macroscopic Pauli scorings. Despite the use of different measurement methods, the IM values for the tibial AC were similar to those reported in the literature (Deneweth et al., 2013; Sim et al., 2014, 2017; Seidenstuecker et al., 2019): while Sim et al. (2014, 2017) and Seidenstuecker et al. (2019) used the same automated indentation mapping method, Deneweth et al. (2013) performed a manual mapping method, where full-thickness 4-mm diameter cylindrical AC explants were extracted from the tibial plateau and exposed to unconfined compression. The IM values were lower in the study of Seidenstuecker et al. (2019), which might be due to the fact that no section-specific subdivision of the tibial plateau was performed during their examinations. While they divided their tibial AC into meniscus-covered and uncovered regions, we additionally grouped the covered parts in the three anatomical regions of the menisci, resulting in more diverging IM results. The values of Deneweth et al. (2013) were generally higher than those reported in any other study, which could be explained by the non-osteoarthritic knees they used in their study and by the different test method. Even so, they observed the same region-specific tendencies for both the meniscus-covered and CtC areas as we saw in the mildly degenerated knee joints. Additionally, Seidenstuecker et al. (2019); Swann and Seedhom (1993), and Yao and Seedhom (1993) measured significantly softer cartilage at the CtC area compared to that covered by the meniscus, which was further underlined by the present study and also found at both the mildly and severely degenerated AC of the tibial plateau.

The *P*_max_ values during the spherical indentation mappings of the knee joint AC and menisci have been investigated very recently (Seidenstuecker et al., 2019; Pordzik et al., 2020a, b) by the same group. In their studies, similar indentation mappings were used: (Pordzik et al., 2020a, b) found mean *P*_max_ values of 0.01 ± 0.01 N for healthy and 0.02 ± 0.02 N for OA menisci. The present study could confirm the previous findings where the severely degenerated menisci exhibited a significantly higher *P*_max_ compared to the mildly degenerated menisci. However, our *P*_max_ values were higher than those of Pordzik et al., which might be explained by the larger indenter diameter (2 vs. 1 mm), deeper indentation depth (0.5 vs. 0.2 mm), and greater indentation speed (0.5 vs. 0.2 mm/s) used in the present study. Seidenstuecker et al. (2019) and Pordzik et al. (2020b) also investigated the *P*_max_ of degenerated and healthy tibial AC. Although using different indenter sizes and indentation parameters, the values of the present study were in the same range as those reported in the literature. However, in contrast to a decrease in the *P*_max_ with progressing degeneration, Seidenstuecker et al. (2019) reported higher *P*_max_ values for their OA cartilage samples compared to healthy samples. Several authors have reported a softening of AC with OA progression (Akizuki et al., 1986; Setton et al., 1999; Temple-Wong et al., 2009; Hosseini et al., 2013), which might help to explain the contrary *P*_max_ findings, which also confirmed the findings of Pordzik et al. (2020b), who found a *P*_max_ decrease with progressing OA.

In the present study, we were unable to combine the time-consuming mapping procedure with a stress relaxation test until the equilibrium state of the AC and menisci was reached. Such relaxation procedures are normally conducted for at least 30–60 min (Korhonen et al., 2002; Chia and Hull, 2008; Martin Seitz et al., 2013; Danso et al., 2017; Warnecke et al., 2020) to obtain the matrix stiffness. However, in the current test setup, such long relaxation times would have resulted in testing times of more than 24 h, which might result in severe autolysis. We would like to point out that the here established *E*_t20_ parameter is rather a measure for viscoelasticity: it is generally accepted that the time-dependent behavior of the AC and meniscus tissue is due to the fluid flow inside the porous matrix. Coluccino et al. (2017) further concluded that the percentage of stress relaxation (Δσ_relax_) can be interpreted as a measure of the viscous properties of the structure that are mainly governed by the proteoglycan content and tissue porosity. During their biomechanical analyses of healthy bovine meniscus samples of different orientations, they identified stress relaxation percentages between 79% and 88% after reaching the equilibrium state (Coluccino et al., 2017). In our study, we found Δσ_relax_ values for mildly and severely degenerated human menisci ranging from 50 to 65% after a relaxation time of 20 s. This reflects the typical negative exponential of the stress relaxation curve of biphasic cartilage and meniscus samples indicating a rapidly decreasing stress. Therefore, we assume that it reflects, to some extent, the viscoelastic behavior of the biphasic structures and allows for a comparison between the different tissues and degeneration groups. Consequently, based on the here obtained parameters IM and *E*_t20_ in combination with Δσ_relax_, it is possible to characterize the viscoelastic properties of the mildly and severely degenerated AC and meniscus tissue.

### Water Content

The water content found in the present study was comparable to the data presented in previous studies, indicating a mean water content ranging from 73.9% for the lateral to 76.4% for the medial mildly degenerated menisci (Table 5). Additionally, regarding the severely degenerated meniscal tissue, we were also able to confirm the results of Herwig et al. (1984); Katsuragawa et al. (2010), Son et al. (2013); Våben et al. (2020), and Warnecke et al. (2020), who found an increased water content in more degenerated menisci. However, similar to the literature, our findings also indicated that this water content increase was neither significant for the medial nor for the lateral menisci.

**TABLE 5:** Mean water content values (in percent) of human knee joint menisci.

Author	*n*_*total*_ lat/med	Lateral	Medial	Comment
Herwig et al., 1984	17	77.1		Water content increased with progressing degeneration
Fithian et al., 1990	54/44	73.8	74.6	
Joshi et al., 1995	5		70.2	
Bursac et al., 2009	33/25	75.4	77.9	Healthy allograft group
Katsuragawa et al., 2010	6/6	75.5	75.1	Healthy
	6/6	76.0	76.5	OA menisci
Martin Seitz et al., 2013	75/75	72.2	73.3	
Son et al., 2013	13/7	79.6	78.0	OA menisci
Danso et al., 2015	13/13	76.8	77.1	
Travascio et al., 2020a	9	70.0		
Våben et al., 2020	7/7	73.0	72.5	Healthy
	43/44	73.0	73.5	OA
Warnecke et al., 2020	24	76.3		Water content increased with progressing degeneration
Morejon et al., 2021	3/5	76.8		
Present study	36/36	73.9	76.4	Mildly degenerated
	36/36	75.0	77.0	Severely degenerated
				

Regarding the water content of the tibial AC, we found an increase of approximately 3% with progressing degeneration. This is in agreement with the findings of Bush and Hall (2003), who found an identical increase in the water content of degenerating tibial AC. In general, the water content in the present study fitted well with the previously published values of human tibial AC (Table 6).

**TABLE 6:** Mean water content values (in percent) of human tibial articular cartilage.

Author	*n*_*total*_		Comment
Treppo et al., 2000	8	79	Healthy
Lusse et al., 2000	19	70.5	OA patients
Bush and Hall, 2003	2	72.3	Tissue grade 1
	3	73.9	Tissue grade 2
	3	76.2	Tissue grade 3
	5	77.7	Tissue grade 4
Silvast et al., 2009	12	73.8	
Travascio et al., 2020b	8	75.0	
Present study	96	74.8	Mildly degenerated
	96	78.1	Severely degenerated

### Limitations

Several factors should be considered when interpreting the results of the present study. Firstly, the preparation technique, particularly the confinement of the menisci using a PMMA cast, might have influenced the biomechanical measurements. As with other *in vitro* indentation setups using cadaveric specimens, this system does not replicate the complex loading behavior of the knee joint, and in particular does not reproduce the circumferential stress induced during physiological loading. Therefore, the here obtained indentation properties of the menisci do not provide bulk properties of the entire tissue and are mainly influenced by the superficial layer and the locally defined region around the indenter. It could be that, at any particular time point when the indentation test was performed, the amount of collagen, fluid, or calcification around the indenter might have influenced the measured biomechanical properties. Even so, during extensive pretesting, we identified a very high reproducibility (>96%) of the identified biomechanical properties.

The setup data to perform the biomechanical mapping, based on the indentation, including the indentation depth and velocity and relaxation time, were not based on physiological strains or loads. Instead, we adapted these setup data from a prior study (Sim et al., 2017) in order to be comparable to the literature. In contrast to the AC mapping setup data, there were no data available in the literature for meniscal mapping. During pretests using both porcine and human menisci, we identified the indentation amplitude and velocity to be the most critical parameters to achieve convergence. Furthermore, the anatomical subregions were based on a standardized method, which did not consider individual anatomy. Firstly, the menisci and meniscus-covered regions at the associated tibial plateau were equally separated into the AH, PI, and PH. This division was based on the middle point between the respective anterior and posterior meniscal attachments. Secondly, the meniscus-covered and uncovered regions were defined in a static position, although the menisci significantly translate on the tibial plateau during knee flexion (Scholes et al., 2015). Both these factors, together with the individual donor variabilities (gender, age, BMI, and knee condition), might explain the deviation in the results between the meniscus-covered regions and the CtC area in the present study.

Another limitation is related to the group grading, which was based on the common clinically used radiographic KL scores. While the KL scores rate the knee as a single entity, it might be that knees were assigned to the severely degenerated group with a severely degenerated medial compartment, whereas the lateral compartment remained in a good condition, potentially leading to a scattered results range within one group. Therefore, it might be beneficial for future studies to divide and group the compartments of each knee joint separately. However, the consecutively conducted macroscopic Pauli scoring of the menisci corroborated the initial degeneration state of the knee joints.

### Conclusion and Outlook

With progressing OA degeneration, we identified a significant increase of both the elastic (IM and *P*_max_) and viscous (*E*_t20_) properties of the lateral and medial menisci, while the relaxation percentage and the water content were not affected. This can be interpreted in a way that the matrix of the menisci becomes stiffer with progressing degeneration while the relaxation behavior seems to be less impaired. In contrast, the elastic properties (IM and *P*_max_) of the adjacent tibial cartilage decreased only in the medial compartment. Therefore, we can conclude that the medial compartment of the here investigated knee joints was much more affected by degenerative changes than the lateral compartment. This coincides with the fact that OA-related medial gonarthrosis is much more frequent than the lateral (Wise et al., 2012). Furthermore, this suggests that the lateral compartments were in an earlier stage of joint degeneration, with the AC still unaffected but the menisci already impaired. Therefore, the results of this biomechanical study suggest that the menisci potentially degenerate earlier than the adjacent AC.

In particular, localization-dependent meniscal stiffening might be attributed to a degeneration-induced crystallization of calcium phosphate within the menisci (Bocher et al., 1965; Hubert et al., 2020). To elucidate the hypothesized crystallization process of the degenerating menisci and their associated role in the degeneration process of the adjacent AC, we are currently working to obtain other knee scores and perform histological analyses and structural quantifications of the collagen and proteoglycan contents. Should our biomechanical findings be reinforced by these additional analyses, the diagnostic paradigms and treatment of OA might be changed, with the focus on the early detection of meniscal degeneration and its respective treatment with the final aim to delay OA onset. Furthermore, the material parameters could be used in combination with finite element programs with automatic optimization subroutines (e.g., simple biphasic model in FEBio) (Maas et al., 2012). This information will be extremely valuable to computational biomechanists and could enrich the current knowledge of the scientific community.

## Data Availability Statement

The raw data supporting the conclusions of this article will be made available by the authors, without undue reservation.

## Ethics Statement

The studies involving human participants were reviewed and approved by IRB No. 70/16, Ulm University, Germany. The patients/participants provided their written informed consent to participate in this study.

## Author Contributions

AS, FO, and JS performed the preparation procedure, mechanical testing, data analysis, and statistics. AS drafted the manuscript. DW assisted in the mechanical testing. MF and MS performed clinical analyses. AI and DW participated in the design and coordination of the study. LD and FO conceived the study and helped in drafting the manuscript. All authors read and approved the final manuscript.

## Conflict of Interest

The authors declare that the research was conducted in the absence of any commercial or financial relationships that could be construed as a potential conflict of interest.

## References

[B1] Akizuki S. Mow V. C. Muller F. Pita J. C. Howell D. S. Manicourt D. H. (1986). Tensile properties of human knee joint cartilage: I. Influence of ionic conditions, weight bearing, and fibrillation on the tensile modulus. *J. Orthop. Res.* 4 379–392. 10.1002/jor.1100040401 3783297

[B2] Andriacchi T. P. Mundermann A. Smith R. L. Alexander E. J. Dyrby C. O. Koo S. (2004). A framework for the in vivo pathomechanics of osteoarthritis at the knee. *Ann. Biomed. Eng.* 32 447–457. 15095819 10.1023/b:abme.0000017541.82498.37

[B3] Bamac B. Ozdemir S. Sarisoy H. T. Colak T. Ozbek A. Akansel G. (2006). Evaluation of medial and lateral meniscus thicknesses in early osteoarthritis of the knee with magnetic resonance imaging. *Saudi Med. J.* 27 854–857. 16758050

[B4] Bhattacharyya T. Gale D. Dewire P. Totterman S. Gale M. E. Mclaughlin S. (2003). The clinical importance of meniscal tears demonstrated by magnetic resonance imaging in osteoarthritis of the knee. *J. Bone Joint Surg. Am.* 85 4–9. 10.2106/00004623-200301000-00002 12533565

[B5] Bocher J. Mankin H. J. Berk R. N. Rodnan G. P. (1965). Prevalence of calcified meniscal cartilage in elderly persons. *N. Engl. J. Med.* 272 1093–1097. 10.1056/nejm196505272722103 14281551

[B6] Buck R. J. Wirth W. Dreher D. Nevitt M. Eckstein F. (2013). Frequency and spatial distribution of cartilage thickness change in knee osteoarthritis and its relation to clinical and radiographic covariates - data from the osteoarthritis initiative. *Osteoarthr. Cartil.* 21 102–109. 10.1016/j.joca.2012.10.010 23099212

[B7] Buck R. J. Wyman B. T. Le Graverand M. P. Hudelmaier M. Wirth W. Eckstein F. (2010). Osteoarthritis may not be a one-way-road of cartilage loss–comparison of spatial patterns of cartilage change between osteoarthritic and healthy knees. *Osteoarthr. Cartil.* 18 329–335. 10.1016/j.joca.2009.11.009 19948267

[B8] Bursac P. York A. Kuznia P. Brown L. M. Arnoczky S. P. (2009). Influence of donor age on the biomechanical and biochemical properties of human meniscal allografts. *Am. J. Sports Med.* 37 884–889. 10.1177/0363546508330140 19336615

[B9] Bush P. G. Hall A. C. (2003). The volume and morphology of chondrocytes within non-degenerate and degenerate human articular cartilage. *Osteoarthr. Cartil.* 11 242–251. 10.1016/s1063-4584(02)00369-2 12681950

[B10] Chia H. N. Hull M. L. (2008). Compressive moduli of the human medial meniscus in the axial and radial directions at equilibrium and at a physiological strain rate. *J. Orthop. Res.* 26 951–956. 10.1002/jor.20573 18271010

[B11] Chu C. R. Williams A. A. Coyle C. H. Bowers M. E. (2012). Early diagnosis to enable early treatment of pre-osteoarthritis. *Arthritis Res. Ther.* 14:212. 10.1186/ar3845 22682469 PMC3446496

[B12] Coluccino L. Peres C. Gottardi R. Bianchini P. Diaspro A. Ceseracciu L. (2017). Anisotropy in the viscoelastic response of knee meniscus cartilage. *J. Appl. Biomater. Funct. Mater.* 15 e77–e83. 27647392 10.5301/jabfm.5000319

[B13] Cooper C. Snow S. Mcalindon T. E. Kellingray S. Stuart B. Coggon D. (2000). Risk factors for the incidence and progression of radiographic knee osteoarthritis. *Arthritis Rheum* 43 995–1000. 10.1002/1529-0131(200005)43:5<995::aid-anr6>3.0.co;2-1 10817551

[B14] Cross W. W. III Saleh K. J. Wilt T. J. Kane R. L. (2006). Agreement about indications for total knee arthroplasty. *Clin. Orthop. Relat. Res.* 446 34–39. 10.1097/01.blo.0000214436.49527.5e 16672869

[B15] Danso E. K. Makela J. T. Tanska P. Mononen M. E. Honkanen J. T. Jurvelin J. S. (2015). Characterization of site-specific biomechanical properties of human meniscus-Importance of collagen and fluid on mechanical nonlinearities. *J. Biomech.* 48 1499–1507. 10.1016/j.jbiomech.2015.01.048 25708321

[B16] Danso E. K. Oinas J. M. T. Saarakkala S. Mikkonen S. Toyras J. Korhonen R. K. (2017). Structure-function relationships of human meniscus. *J. Mech. Behav. Biomed. Mater.* 67 51–60. 27987426 10.1016/j.jmbbm.2016.12.002

[B17] Deneweth J. M. Newman K. E. Sylvia S. M. Mclean S. G. Arruda E. M. (2013). Heterogeneity of tibial plateau cartilage in response to a physiological compressive strain rate. *J. Orthop. Res.* 31 370–375. 10.1002/jor.22226 22952052

[B18] Down C. Xu Y. Osagie L. E. Bostrom M. P. G. (2011). The lack of correlation between radiographic findings and cartilage integrity. *J. Arthroplast.* 26 949–954. 10.1016/j.arth.2010.09.007 21144698

[B19] Ebrahimi M. Turunen M. J. Finnila M. A. Joukainen A. Kroger H. Saarakkala S. (2020). Structure-function relationships of healthy and osteoarthritic human tibial cartilage: experimental and numerical investigation. *Ann. Biomed. Eng.* 48 2887–2900. 10.1007/s10439-020-02559-0 32648191 PMC7723942

[B20] Englund M. (2009). The role of the meniscus in osteoarthritis genesis. *Med. Clin. North Am.* 93 37–43. 10.1016/j.mcna.2008.08.005 19059020

[B21] Englund M. (2010). The role of biomechanics in the initiation and progression of OA of the knee. *Best Pract. Res. Clin. Rheumatol.* 24 39–46. 10.1016/j.berh.2009.08.008 20129198

[B22] Englund M. Guermazi A. Lohmander S. L. (2009). The role of the meniscus in knee osteoarthritis: a cause or consequence? *Radiol. Clin. North Am.* 47 703–712. 10.1016/j.rcl.2009.03.003 19631077

[B23] Englund M. Roemer F. W. Hayashi D. Crema M. D. Guermazi A. (2012). Meniscus pathology, osteoarthritis and the treatment controversy. *Nat. Rev. Rheumatol.* 8 412–419. 10.1038/nrrheum.2012.69 22614907

[B24] Erbagci H. Gumusburun E. Bayram M. Karakurum G. Sirikci A. (2004). The normal menisci: in vivo MRI measurements. *Surg. Radiol. Anat.* 26 28–32. 10.1007/s00276-003-0182-2 14574467

[B25] Faber S. C. Eckstein F. Lukasz S. Muhlbauer R. Hohe J. Englmeier K. H. (2001). Gender differences in knee joint cartilage thickness, volume and articular surface areas: assessment with quantitative three-dimensional MR imaging. *Skeletal. Radiol.* 30 144–150. 10.1007/s002560000320 11357452

[B26] Faul F. Erdfelder E. Lang A. G. Buchner A. (2007). G^∗^Power 3: a flexible statistical power analysis program for the social, behavioral, and biomedical sciences. *Behav. Res. Methods* 39 175–191. 10.3758/bf03193146 17695343

[B27] Fischenich K. M. Lewis J. Kindsfater K. A. Bailey T. S. Haut Donahue T. L. (2015). Effects of degeneration on the compressive and tensile properties of human meniscus. *J. Biomech.* 48 1407–1411. 10.1016/j.jbiomech.2015.02.042 25770751

[B28] Fithian D. C. Kelly M. A. Mow V. C. (1990). Material properties and structure-function relationships in the menisci. *Clin. Orthop. Relat. Res.* 252 19–31. 2406069

[B29] Fox A. J. Bedi A. Rodeo S. A. (2012). The basic science of human knee menisci: structure, composition, and function. *Sports Health* 4 340–351. 10.1177/1941738111429419 23016106 PMC3435920

[B30] Guilak F. (2011). Biomechanical factors in osteoarthritis. *Best Pract. Res. Clin. Rheumatol.* 25 815–823. 10.1016/j.berh.2011.11.013 22265263 PMC3266544

[B31] Hadjab I. Sim S. Karhula S. S. Kauppinen S. Garon M. Quenneville E. (2018). Electromechanical properties of human osteoarthritic and asymptomatic articular cartilage are sensitive and early detectors of degeneration. *Osteoarthr. Cartil.* 26 405–413. 10.1016/j.joca.2017.12.002 29229562

[B32] Hayes W. C. Keer L. M. Herrmann G. Mockros L. F. (1972). A mathematical analysis for indentation tests of articular cartilage. *J. Biomech.* 5 541–551. 10.1016/0021-9290(72)90010-3 4667277

[B33] Herwig J. Egner E. Buddecke E. (1984). Chemical-changes of human knee-joint menisci in various stages of degeneration. *Ann. Rheum. Dis.* 43 635–640. 10.1136/ard.43.4.635 6548109 PMC1001426

[B34] Hochberg M. C. Lethbridge-Cejku M. Scott W. W. Jr. Reichle R. Plato C. C. Tobin J. D. (1995). The association of body weight, body fatness and body fat distribution with osteoarthritis of the knee: data from the Baltimore Longitudinal Study of Aging. *J. Rheumatol.* 22 488–493. 7783067

[B35] Horbert V. Lange M. Reuter T. Hoffmann M. Bischoff S. Borowski J. (2019). Comparison of near-infrared spectroscopy with needle indentation and histology for the determination of cartilage thickness in the large animal model sheep. *Cartilage* 10 173–185. 10.1177/1947603517731851 28980486 PMC6425542

[B36] Hosseini S. M. Veldink M. B. Ito K. Van Donkelaar C. (2013). Is collagen fiber damage the cause of early softening in articular cartilage? *Osteoarthr. Cartil.* 21 136–143. 10.1016/j.joca.2012.09.002 23010079

[B37] Hubert J. Beil F. T. Rolvien T. Butscheidt S. Hischke S. Puschel K. (2020). Cartilage calcification is associated with histological degeneration of the knee joint: a highly prevalent, age-independent systemic process. *Osteoarthr. Cartil.* 28 1351–1361. 10.1016/j.joca.2020.04.020 32683044

[B38] Hunter D. J. Niu J. B. Zhang Y. Lavalley M. Mclennan C. E. Hudelmaier M. (2008). Premorbid knee osteoarthritis is not characterised by diffuse thinness: the Framingham Osteoarthritis Study. *Ann. Rheum. Dis.* 67 1545–1549. 10.1136/ard.2007.076810 18218668 PMC3653621

[B39] Hunter D. J. Zhang Y. Q. Niu J. B. Tu X. Amin S. Clancy M. (2006). The association of meniscal pathologic changes with cartilage loss in symptomatic knee osteoarthritis. *Arthritis Rheum.* 54 795–801. 10.1002/art.21724 16508930

[B40] Jackson B. D. Wluka A. E. Teichtahl A. J. Morris M. E. Cicuttini F. M. (2004). Reviewing knee osteoarthritis–a biomechanical perspective. *J. Sci. Med. Sport* 7 347–357. 10.1016/s1440-2440(04)80030-6 15518300

[B41] Joshi M. D. Suh J. K. Marui T. Woo S. L. (1995). Interspecies variation of compressive biomechanical properties of the meniscus. *J. Biomed. Mater. Res.* 29 823–828. 10.1002/jbm.820290706 7593020

[B42] Jurvelin J. S. Rasanen T. Kolmonen P. Lyyra T. (1995). Comparison of optical, needle probe and ultrasonic techniques for the measurement of articular cartilage thickness. *J. Biomech.* 28 231–235. 10.1016/0021-9290(94)00060-h 7896866

[B43] Katsuragawa Y. Saitoh K. Tanaka N. Wake M. Ikeda Y. Furukawa H. (2010). Changes of human menisci in osteoarthritic knee joints. *Osteoarthr. Cartil.* 18 1133–1143. 10.1016/j.joca.2010.05.017 20633672

[B44] Kellgren J. H. Lawrence J. S. (1957). Radiological assessment of osteo-arthrosis. *Ann. Rheum. Dis.* 16 494–502. 10.1136/ard.16.4.494 13498604 PMC1006995

[B45] Korhonen R. K. Laasanen M. S. Toyras J. Rieppo J. Hirvonen J. Helminen H. J. (2002). Comparison of the equilibrium response of articular cartilage in unconfined compression, confined compression and indentation. *J. Biomech.* 35 903–909. 10.1016/s0021-9290(02)00052-0 12052392

[B46] Kwok J. Grogan S. Meckes B. Arce F. Lal R. D’lima D. (2014). Atomic force microscopy reveals age-dependent changes in nanomechanical properties of the extracellular matrix of native human menisci: implications for joint degeneration and osteoarthritis. *Nanomedicine* 10 1777–1785. 10.1016/j.nano.2014.06.010 24972006 PMC4374607

[B47] Loeser R. F. Goldring S. R. Scanzello C. R. Goldring M. B. (2012). Osteoarthritis: a disease of the joint as an organ. *Arthritis Rheum.* 64 1697–1707. 10.1002/art.34453 22392533 PMC3366018

[B48] Lohmander L. S. Englund P. M. Dahl L. L. Roos E. M. (2007). The long-term consequence of anterior cruciate ligament and meniscus injuries: osteoarthritis. *Am. J. Sports Med.* 35 1756–1769. 10.1177/0363546507307396 17761605

[B49] Lusse S. Claassen H. Gehrke T. Hassenpflug J. Schunke M. Heller M. (2000). Evaluation of water content by spatially resolved transverse relaxation times of human articular cartilage. *Magn. Reson. Imaging* 18 423–430. 10.1016/s0730-725x(99)00144-7 10788720

[B50] Maas S. A. Ellis B. J. Ateshian G. A. Weiss J. A. (2012). FEBio: finite elements for biomechanics. *J. Biomech. Eng.* 134:011005. 10.1115/1.4005694 PMC3705975 22482660

[B51] Mainil-Varlet P. Aigner T. Brittberg M. Bullough P. Hollander A. Hunziker E. (2003). Histological assessment of cartilage repair: a report by the histology endpoint committee of the international cartilage repair society (ICRS). *J. Bone Joint Surg. Am.* 85-A (Suppl. 2) 45–57. 10.2106/00004623-200300002-00007 12721345

[B52] Mankin H. J. Johnson M. E. Lippiello L. (1981). Biochemical and metabolic abnormalities in articular cartilage from osteoarthritic human hips. III. Distribution and metabolism of amino sugar-containing macromolecules. *J. Bone Joint Surg. Am.* 63 131–139. 10.2106/00004623-198163010-00017 7451514

[B53] Marchiori G. Berni M. Boi M. Filardo G. (2019). Cartilage mechanical tests: evolution of current standards for cartilage repair and tissue engineering. A literature review. *Clin. Biomech. (Bristol, Avon)* 68 58–72. 10.1016/j.clinbiomech.2019.05.019 31158591

[B54] Martin Seitz A. Galbusera F. Krais C. Ignatius A. Durselen L. (2013). Stress-relaxation response of human menisci under confined compression conditions. *J. Mech. Behav. Biomed. Mater.* 26 68–80. 10.1016/j.jmbbm.2013.05.027 23811278

[B55] Morejon A. Norberg C. D. De Rosa M. Best T. M. Jackson A. R. Travascio F. (2021). Compressive properties and hydraulic permeability of human meniscus: relationships with tissue structure and composition. *Front. Bioeng. Biotechnol.* 8:622552. 10.3389/fbioe.2020.622552 33644008 PMC7902918

[B56] Moyer J. T. Abraham A. C. Haut Donahue T. L. (2012). Nanoindentation of human meniscal surfaces. *J. Biomech.* 45 2230–2235. 10.1016/j.jbiomech.2012.06.017 22789734 PMC3422427

[B57] Moyer J. T. Priest R. Bouman T. Abraham A. C. Donahue T. L. (2013). Indentation properties and glycosaminoglycan content of human menisci in the deep zone. *Acta Biomater.* 9 6624–6629. 10.1016/j.actbio.2012.12.033 23321302 PMC3628809

[B58] Neogi T. (2013). The epidemiology and impact of pain in osteoarthritis. *Osteoarthr. Cartil.* 21 1145–1153. 10.1016/j.joca.2013.03.018 23973124 PMC3753584

[B59] Park J. Y. Kim J. K. Cheon J. E. Lee M. C. Han H. S. (2020). Meniscus stiffness measured with shear wave elastography is correlated with meniscus degeneration. *Ultrasound Med. Biol.* 46 297–304. 10.1016/j.ultrasmedbio.2019.10.014 31753598

[B60] Pauli C. Grogan S. P. Patil S. Otsuki S. Hasegawa A. Koziol J. (2011). Macroscopic and histopathologic analysis of human knee menisci in aging and osteoarthritis. *Osteoarthr. Cartil.* 19 1132–1141. 10.1016/j.joca.2011.05.008 21683797 PMC3217905

[B61] Pflieger I. Stolberg-Stolberg J. Foehr P. Kuntz L. Tubel J. Grosse C. U. (2019). Full biomechanical mapping of the ovine knee joint to determine creep-recovery, stiffness and thickness variation. *Clin. Biomech. (Bristol, Avon)* 67 1–7. 10.1016/j.clinbiomech.2019.04.015 31054436

[B62] Pordzik J. Bernstein A. Mayr H. O. Latorre S. H. Maks A. Schmal H. (2020a). Analysis of proteoglycan content and biomechanical properties in arthritic and arthritis-free menisci. *Appl. Sci. Basel* 10:9012. 10.3390/app10249012

[B63] Pordzik J. Bernstein A. Watrinet J. Mayr H. O. Latorre S. H. Schmal H. (2020b). Correlation of biomechanical alterations under gonarthritis between overlying menisci and articular cartilage. *Appl. Sci. Basel* 10:8673. 10.3390/app10238673

[B64] Ruiz D. Jr. Koenig L. Dall T. M. Gallo P. Narzikul A. Parvizi J. (2013). The direct and indirect costs to society of treatment for end-stage knee osteoarthritis. *J. Bone Joint Surg. Am.* 95 1473–1480. 10.2106/jbjs.l.01488 23965697

[B65] Ryd L. Brittberg M. Eriksson K. Jurvelin J. S. Lindahl A. Marlovits S. (2015). Pre-Osteoarthritis: definition and diagnosis of an elusive clinical entity. *Cartilage* 6 156–165. 10.1177/1947603515586048 26175861 PMC4481392

[B66] Scholes C. Houghton E. R. Lee M. Lustig S. (2015). Meniscal translation during knee flexion: what do we really know? *Knee Surg. Sports Traumatol. Arthrosc.* 23 32–40. 10.1007/s00167-013-2482-3 23568385

[B67] Seidenstuecker M. Watrinet J. Bernstein A. Suedkamp N. P. Latorre S. H. Maks A. (2019). Viscoelasticity and histology of the human cartilage in healthy and degenerated conditions of the knee. *J. Orthop. Surg. Res.* 14:256. 10.1186/s13018-019-1308-5 PMC6693159 31409382

[B68] Setton L. A. Elliott D. M. Mow V. C. (1999). Altered mechanics of cartilage with osteoarthritis: human osteoarthritis and an experimental model of joint degeneration. *Osteoarthr. Cartil.* 7 2–14. 10.1053/joca.1998.0170 10367011

[B69] Silvast T. S. Jurvelin J. S. Lammi M. J. Toyras J. (2009). pQCT study on diffusion and equilibrium distribution of iodinated anionic contrast agent in human articular cartilage–associations to matrix composition and integrity. *Osteoarthr. Cartil.* 17 26–32. 10.1016/j.joca.2008.05.012 18602844

[B70] Sim S. Chevrier A. Garon M. Quenneville E. Lavigne P. Yaroshinsky A. (2017). Electromechanical probe and automated indentation maps are sensitive techniques in assessing early degenerated human articular cartilage. *J. Orthop. Res.* 35 858–867. 10.1002/jor.23330 27279435

[B71] Sim S. Chevrier A. Garon M. Quenneville E. Yaroshinsky A. Hoemann C. D. (2014). Non-destructive electromechanical assessment (Arthro-BST) of human articular cartilage correlates with histological scores and biomechanical properties. *Osteoarthr. Cartil.* 22 1926–1935. 10.1016/j.joca.2014.08.008 25168362

[B72] Son M. Goodman S. B. Chen W. Hargreaves B. A. Gold G. E. Levenston M. E. (2013). Regional variation in T1rho and T2 times in osteoarthritic human menisci: correlation with mechanical properties and matrix composition. *Osteoarthr. Cartil.* 21 796–805. 10.1016/j.joca.2013.03.002 23499673 PMC3909565

[B73] Stefanik J. J. Guermazi A. Roemer F. W. Peat G. Niu J. Segal N. A. (2016). Changes in patellofemoral and tibiofemoral joint cartilage damage and bone marrow lesions over 7 years: the Multicenter Osteoarthritis Study. *Osteoarthr. Cartil.* 24 1160–1166. 10.1016/j.joca.2016.01.981 26836287 PMC4907825

[B74] Swann A. C. Seedhom B. B. (1993). The stiffness of normal articular cartilage and the predominant acting stress levels: implications for the aetiology of osteoarthrosis. *Br. J. Rheumatol.* 32 16–25. 10.1093/rheumatology/32.1.16 8422553

[B75] Takroni T. Laouar L. Adesida A. Elliott J. A. Jomha N. M. (2016). Anatomical study: comparing the human, sheep and pig knee meniscus. *J. Exp. Orthop.* 3:35. 10.1186/s40634-016-0071-3 PMC5143332 27928740

[B76] Temple-Wong M. M. Bae W. C. Chen M. Q. Bugbee W. D. Amiel D. Coutts R. D. (2009). Biomechanical, structural, and biochemical indices of degenerative and osteoarthritic deterioration of adult human articular cartilage of the femoral condyle. *Osteoarthr. Cartil.* 17 1469–1476. 10.1016/j.joca.2009.04.017 19464244 PMC2763930

[B77] Travascio F. Devaux F. Volz M. Jackson A. R. (2020a). Molecular and macromolecular diffusion in human meniscus: relationships with tissue structure and composition. *Osteoarthr. Cartil.* 28 375–382. 10.1016/j.joca.2019.12.006 31917232 PMC7248550

[B78] Travascio F. Valladares-Prieto S. Jackson A. R. (2020b). Effects of solute size and tissue composition on molecular and macromolecular diffusivity in human knee cartilage. *Osteoarthr. Cartil. Open* 2:100087. 10.1016/j.ocarto.2020.100087 PMC8489571 34611626

[B79] Treppo S. Koepp H. Quan E. C. Cole A. A. Kuettner K. E. Grodzinsky A. J. (2000). Comparison of biomechanical and biochemical properties of cartilage from human knee and ankle pairs. *J. Orthop. Res.* 18 739–748. 10.1002/jor.1100180510 11117295

[B80] Tsujii A. Nakamura N. Horibe S. (2017). Age-related changes in the knee meniscus. *Knee* 24 1262–1270. 10.1016/j.knee.2017.08.001 28970119

[B81] Våben C. Heinemeier K. M. Schjerling P. Olsen J. Petersen M. M. Kjaer M. (2020). No detectable remodelling in adult human menisci: an analysis based on the C14 bomb pulse. *Br. J. Sports Med.* 54 1433–1437. 10.1136/bjsports-2019-101360 32409517 PMC7677461

[B82] Waldstein W. Perino G. Gilbert S. L. Maher S. A. Windhager R. Boettner F. (2016). OARSI osteoarthritis cartilage histopathology assessment system: a biomechanical evaluation in the human knee. *J. Orthop. Res.* 34 135–140. 10.1002/jor.23010 26250350

[B83] Warnecke D. Balko J. Haas J. Bieger R. Leucht F. Wolf N. (2020). Degeneration alters the biomechanical properties and structural composition of lateral human menisci. *Osteoarthr. Cartil.* 28 1482–1491. 10.1016/j.joca.2020.07.004 32739340

[B84] Willinger L. Foehr P. Achtnich A. Forkel P. Voss A. Liska F. (2019). Effect of lower limb alignment in medial meniscus-deficient knees on tibiofemoral contact pressure. *Orthop. J. Sports Med.* 7:2325967118824611. 10.1177/2325967118824611 PMC6378645 30800688

[B85] Wise B. L. Niu J. Yang M. Lane N. E. Harvey W. Felson D. T. (2012). Patterns of compartment involvement in tibiofemoral osteoarthritis in men and women and in whites and African Americans. *Arthritis Care Res. (Hoboken)* 64 847–852. 10.1002/acr.21606 22238208 PMC3340516

[B86] Yao J. Q. Seedhom B. B. (1993). Mechanical conditioning of articular cartilage to prevalent stresses. *Br. J. Rheumatol.* 32 956–965. 10.1093/rheumatology/32.11.956 8220934

