# Supplementary material for: Osteoarthritis-Related Degeneration Alters the Biomechanical Properties of Human Menisci Before the Articular Cartilage
Source: Front Bioeng Biotechnol. 2021 May 6;9:659989. doi: 10.3389/fbioe.2021.659989 (PMC8134692; doi:10.3389/fbioe.2021.659989)

Instantaneous modulus in MPa (Mild degenerated 1-6)

AC Femur | AC Tibia | Lateral Meniscus | Medial Meniscus

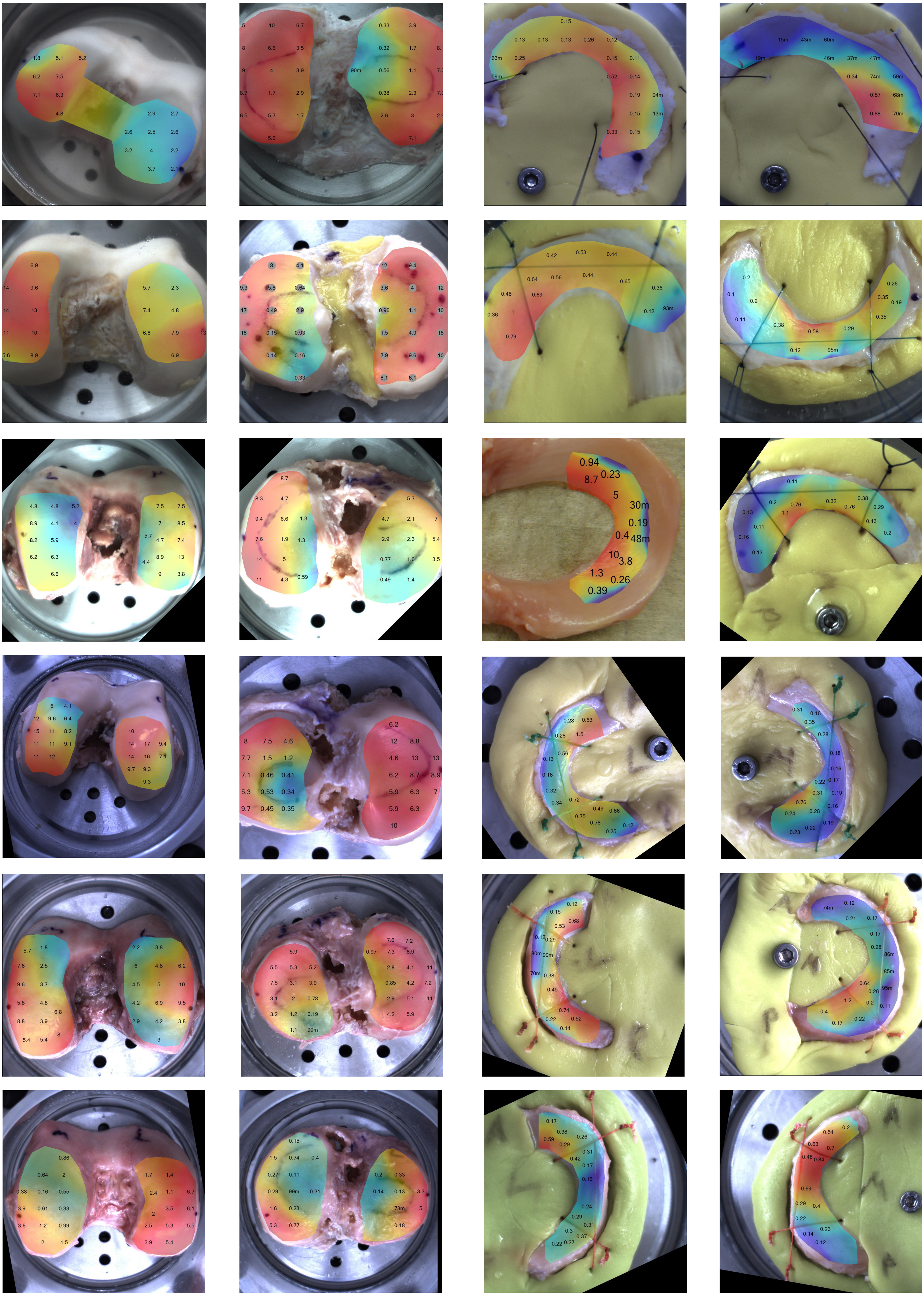

Instantaneous modulus in MPa (Mild degenerated 7-12)

AC Femur	AC Tibia	Lateral Meniscus	Medial Meniscus

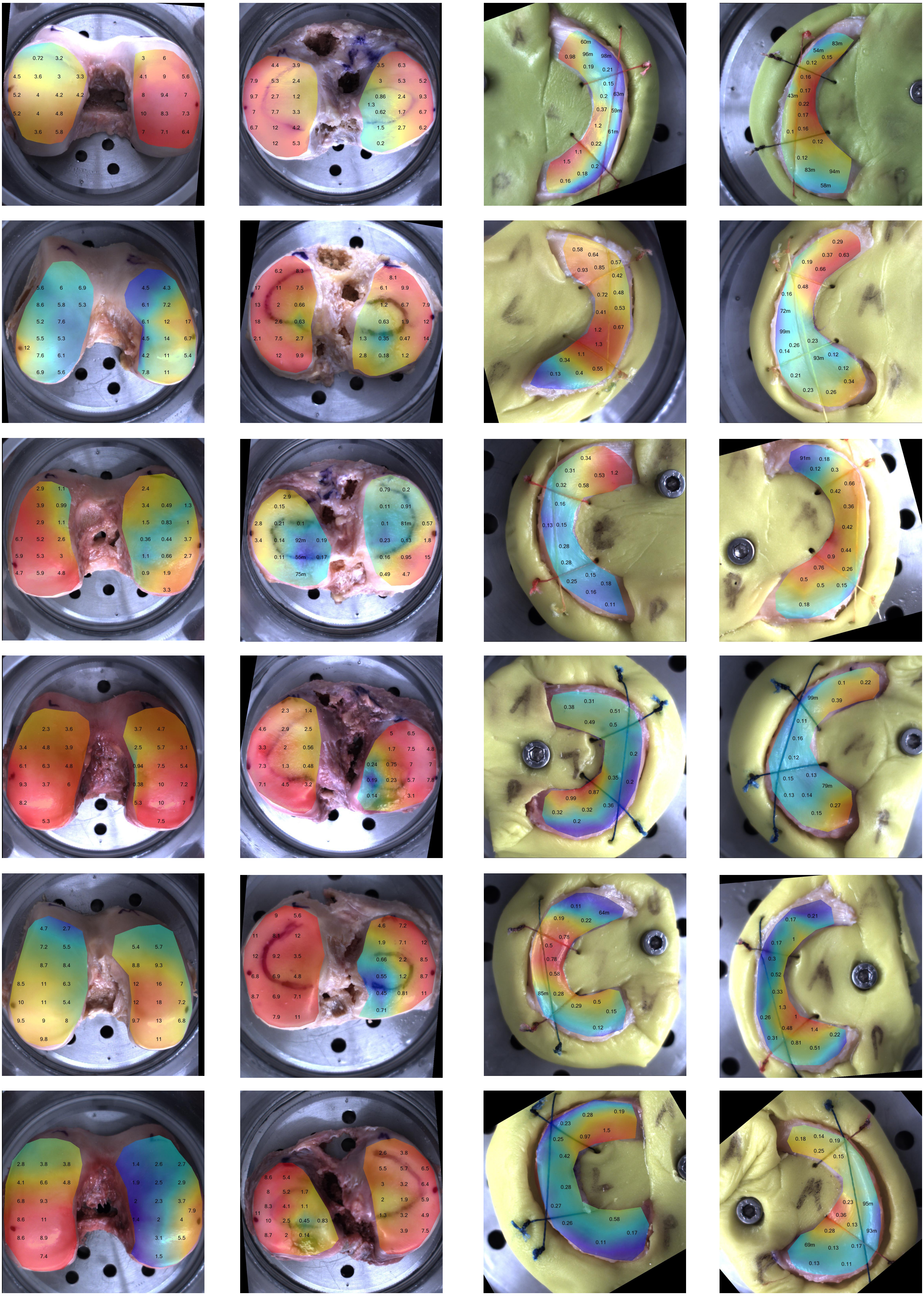

Instantaneous modulus in MPa (Severe degenerated 1-6)

AC Femur | AC Tibia | Lateral Meniscus | Medial Meniscus

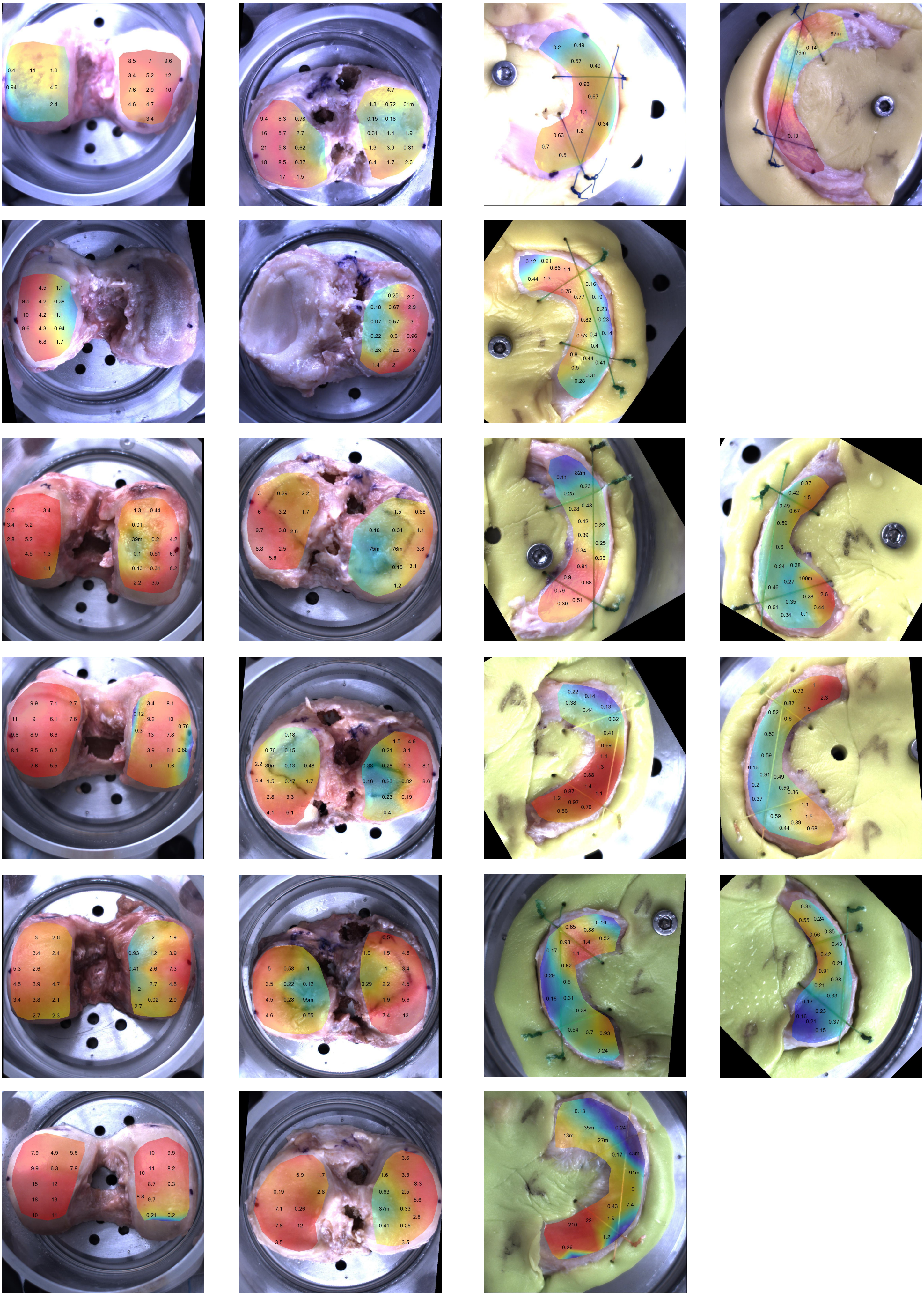

Instantaneous modulus in MPa (Severe degenerated 7-12)

AC Femur | AC Tibia | Lateral Meniscus | Medial Meniscus

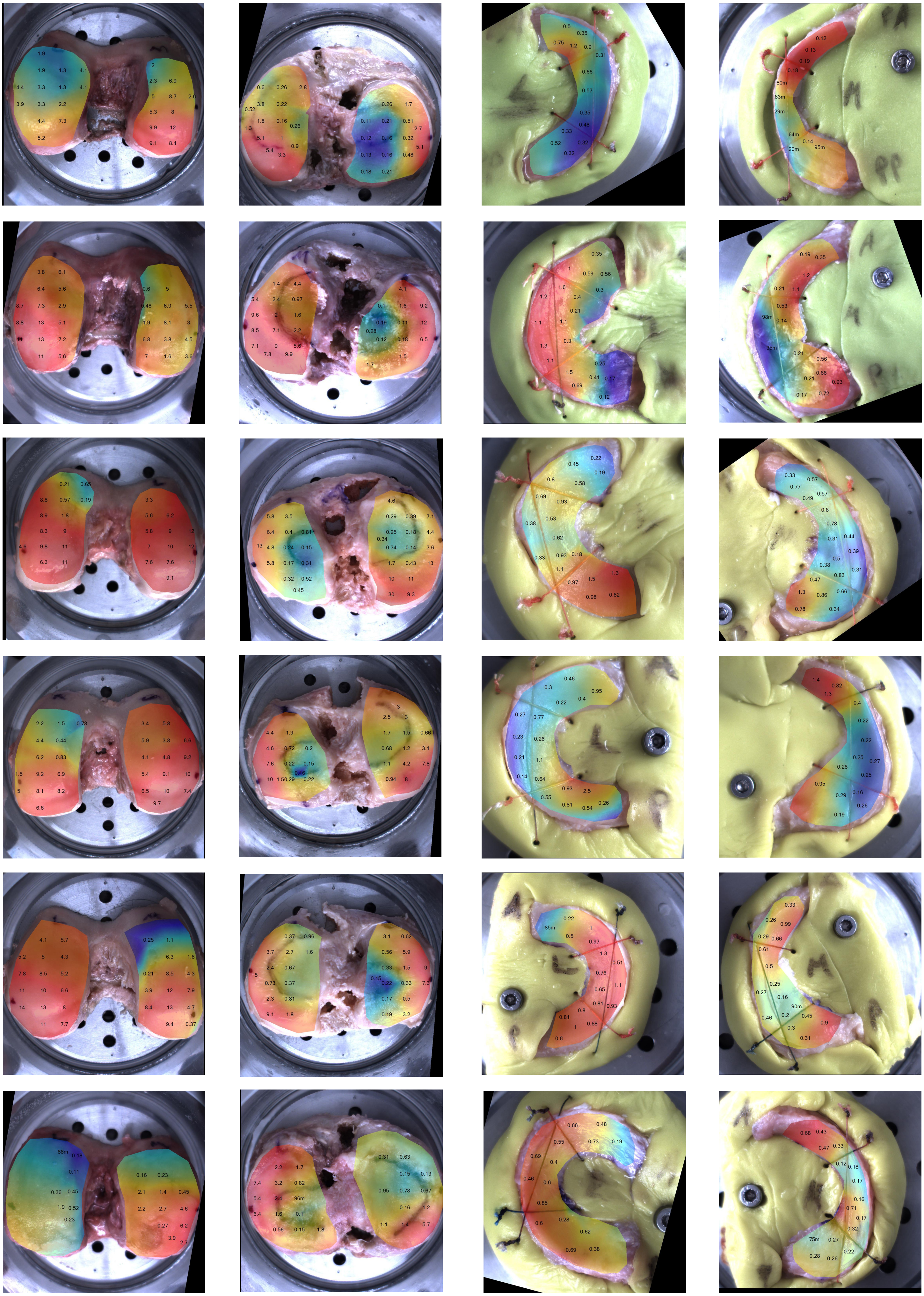

Supplement: Supplementary file 1 [file Data_Sheet_1.PDF]
