# Supplementary material for: Osteoarthritis-Related Degeneration Alters the Biomechanical Properties of Human Menisci Before the Articular Cartilage
Source: Front Bioeng Biotechnol. 2021 May 6;9:659989. doi: 10.3389/fbioe.2021.659989 (PMC8134692; doi:10.3389/fbioe.2021.659989)

# Thickness in mm (Mild degenerated 1-6)

AC Femur | AC Tibia | Lateral Meniscus | Medial Meniscus

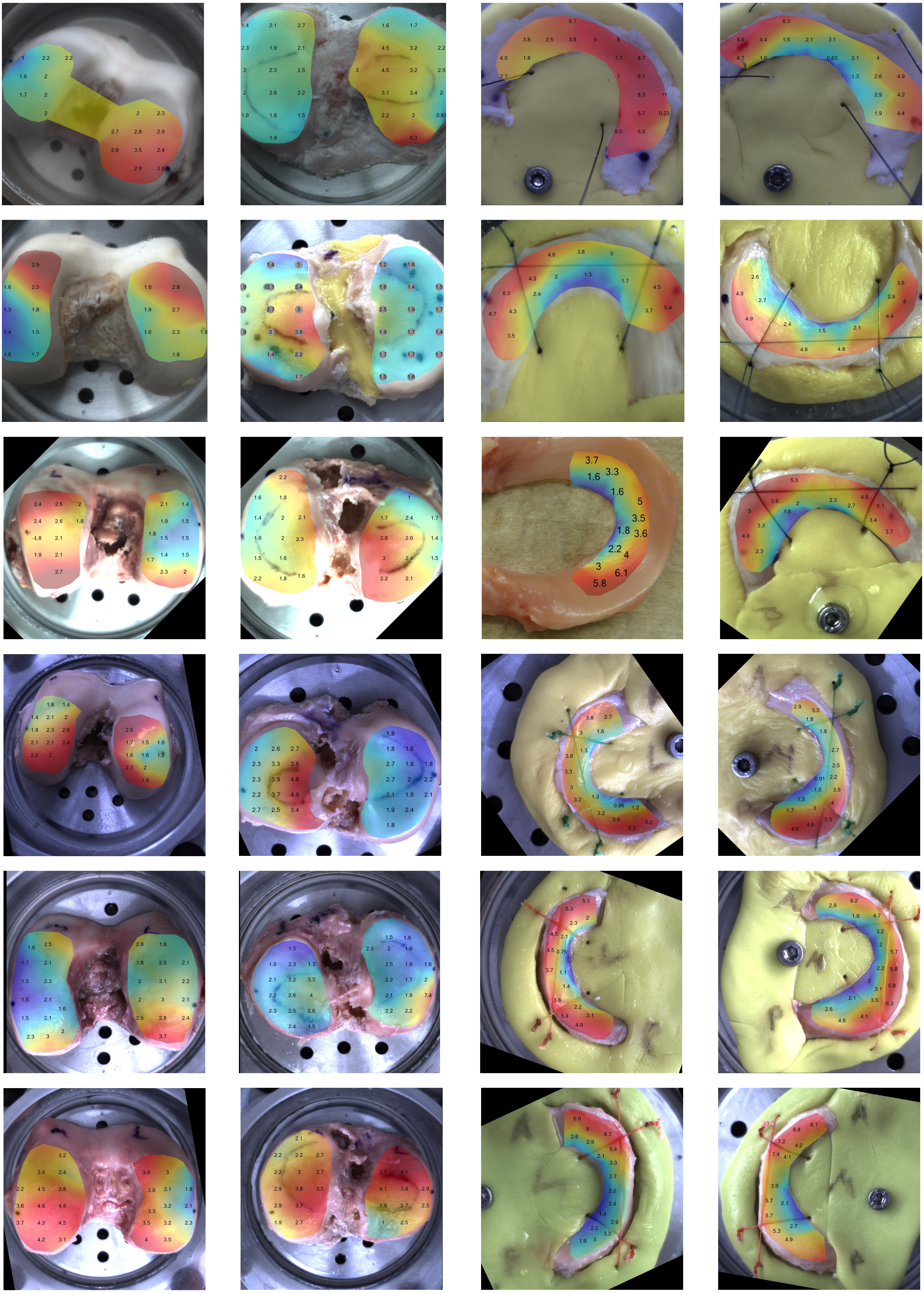

## Thickness in mm (Mild degenerated 7-12)

AC Femur | AC Tibia | Lateral Meniscus | Medial Meniscus

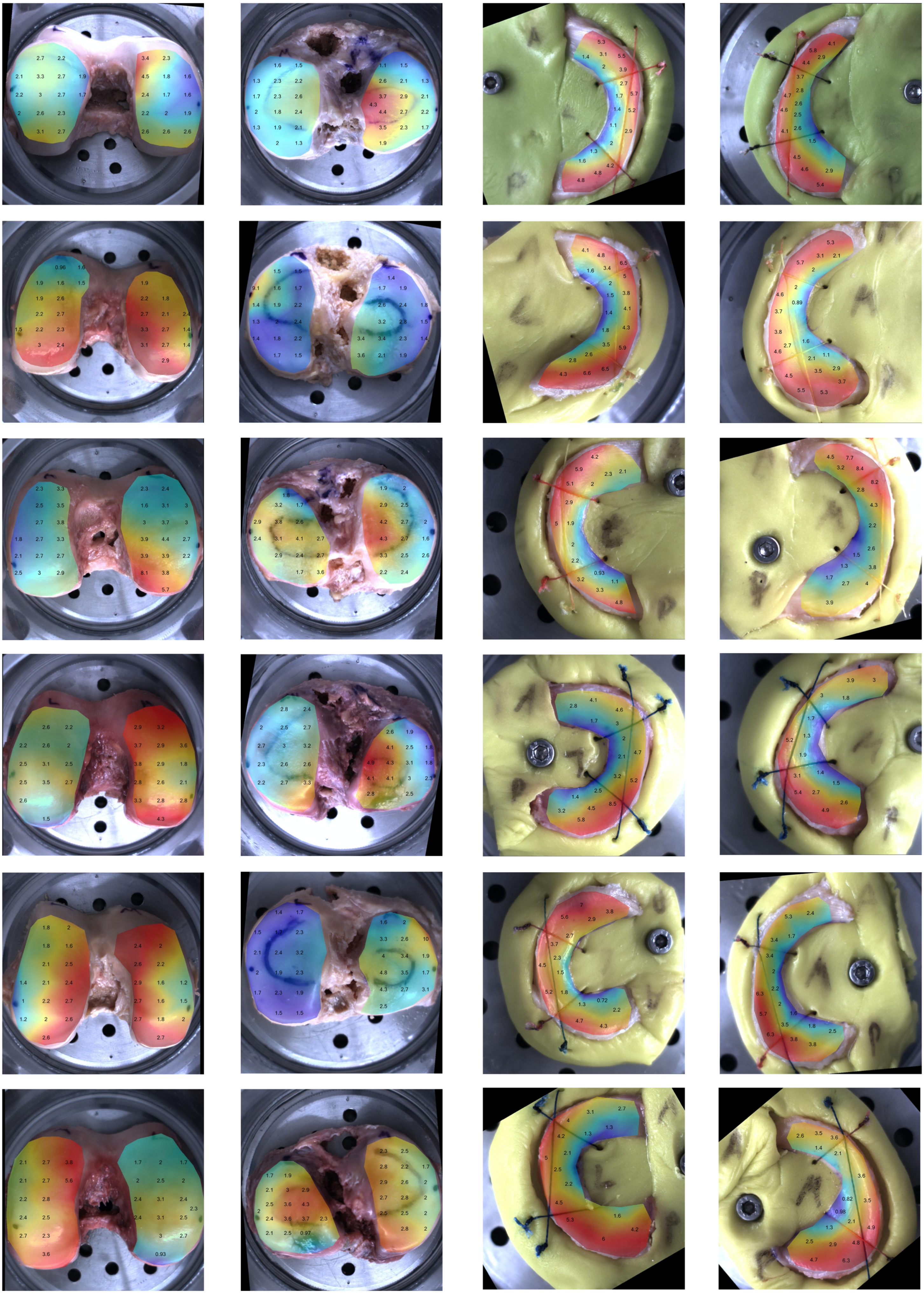

## Thickness in mm (Severe degenerated 1-6)

AC Femur | AC Tibia | Lateral Meniscus | Medial Meniscus

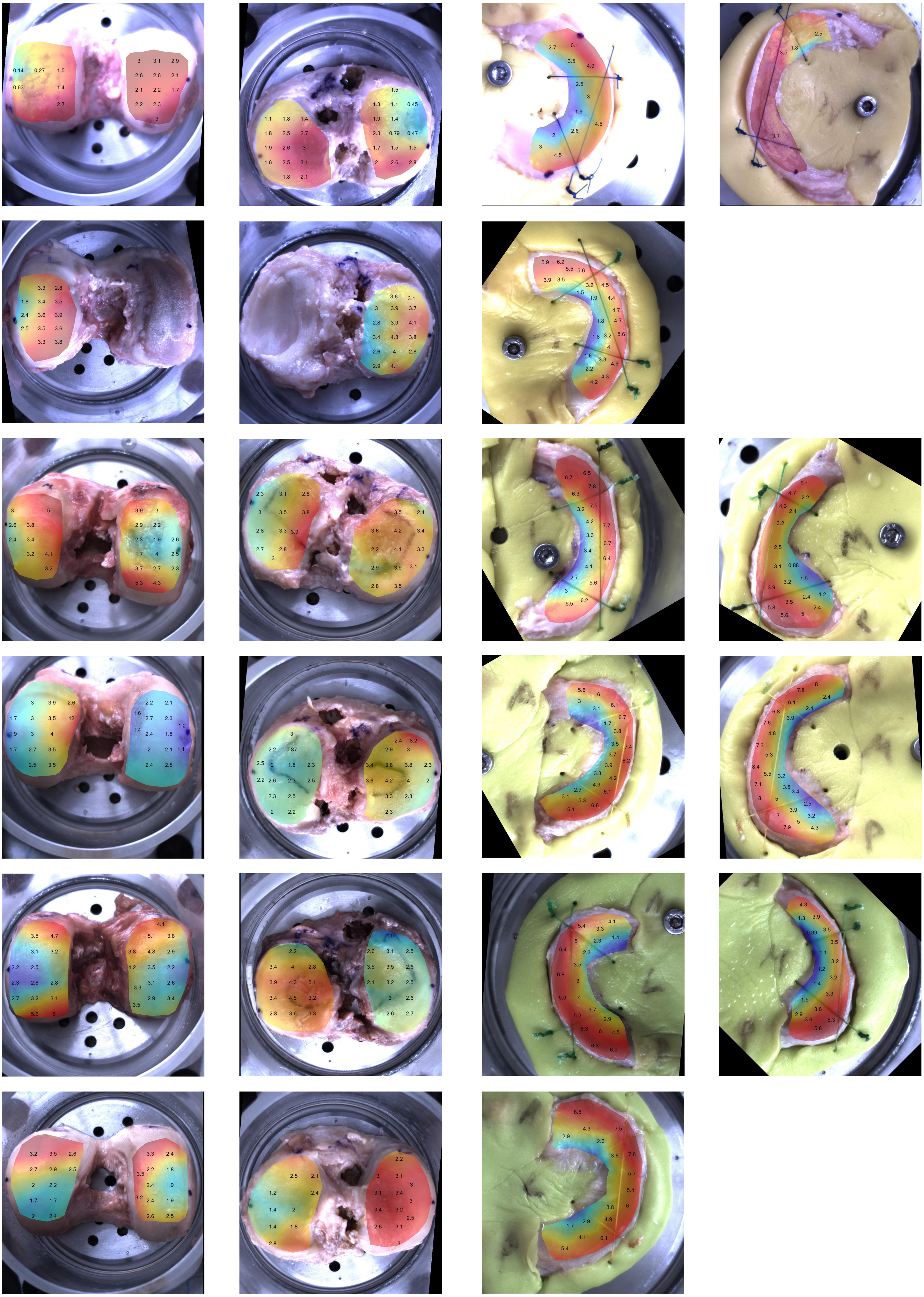

## Thickness in mm (Severe degenerated 7-12)

| AC Femur | AC Tibia | Lateral Meniscus | Medial Meniscus |
|---|---|---|---|

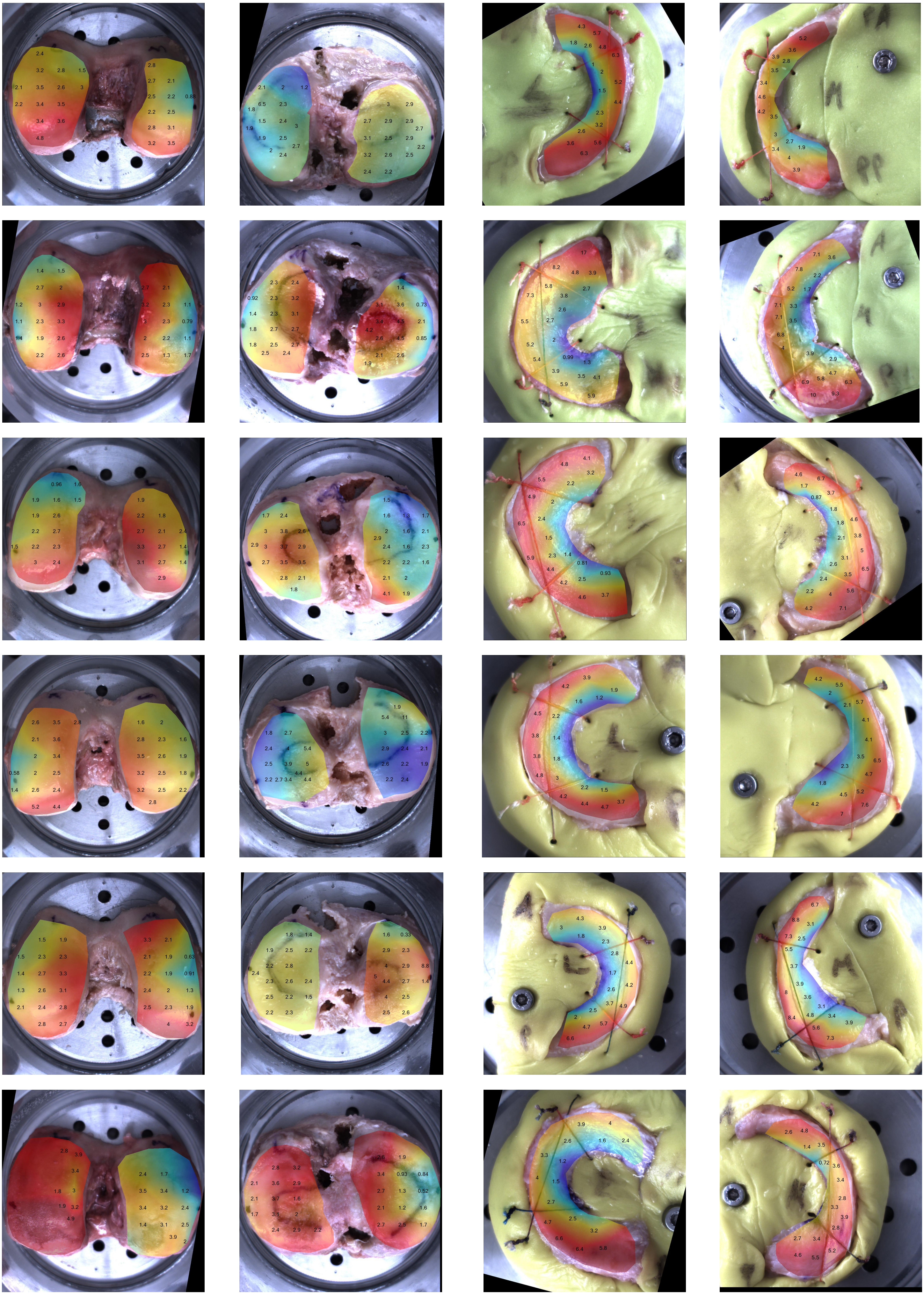

Supplement: Supplementary file 2 [file Data_Sheet_2.PDF]
